# Transmission of Zoonotic Diseases in the Daily Life of Ancient *Pompeii* and *Herculaneum* (79 CE, Italy): A Review of Animal–Human–Environment Interactions through Biological, Historical and Archaeological Sources

**DOI:** 10.3390/ani12020213

**Published:** 2022-01-17

**Authors:** Carmen Tanga, Marta Remigio, Joan Viciano

**Affiliations:** 1 Department of Legal Medicine, Toxicology and Physical Anthropology, Faculty of Medicine, University of Granada, Avenida de la Investigación 11, 18071 Granada, Spain; carmentanga@correo.ugr.es; 2 Independent Researcher, Strada Fonte Borea 1, 65125 Pescara, Italy; martaire3@gmail.com; 3 Department of Medicine and Ageing Sciences, ‘G. d’Annunzio’ University of Chieti-Pescara, Via L. Polacchi 11-13, 66100 Chieti, Italy

**Keywords:** zoonosis, human–animal interaction, zooarchaeology, archaeology, palaeopathology, physical anthropology, habits and lifestyle, status of health and disease, *Pompeii* and *Herculaneum*

## Abstract

**Simple Summary:**

There is a wide range of historical, archaeological and biological sources to help understand the nature of health and disease in the ancient Roman world. In this study, we examine various predisposing elements typical of the urban environment in relation to zoonotic diseases in the ancient Roman cities of *Pompeii* and *Herculaneum*, both devastated by pyroclastic surges as a consequence of the eruption of Mount Somma–Vesuvius in 79 CE. We analyse the meaning and value of the sources to develop an understanding of the many features of everyday life in these cities. We make judgements about issues relevant to the reconstruction of the past, and finally, we synthesize evidence from different sources to construct historical explanations and arguments in relation to the transmission of zoonotic diseases in these ancient Roman populations.

**Abstract:**

There is no doubt that the cultural and urban environments contributed to the animal–human interaction in the daily life of the ancient Roman world. The singularity of the circumstances of the burial of *Pompeii* and *Herculaneum*, together with literary sources and the extraordinary state of preservation of the archaeological and biological material found, has provided researchers with an opportunity, unique in its kind, to reconstruct the life and ways of living of its inhabitants. This study illustrates the main drivers and mechanisms for the distribution and transmission of zoonotic diseases in these ancient Roman populations, such as (i) the large number and role that different animal species played in the ancient Roman world; (ii) the environmental conditions for the survival of parasites, pathogens and vectors; (iii) the great variety and intensity of commercial activities and occupations that presented certain risks of infections; (iv) the absence of adequate safety controls during processing, distribution and preservation of foodstuffs in unsuitable environments and some culinary habits; (v) the inadequate mechanisms of the disposal of human waste and the biotic contamination of watercourses and reservoirs; and finally (vi) the use of animals related to religious and cultural practices.

## 1. Introduction

Zoonosis (from ancient Greek *ζῷον* [*zôion*, animal] and *νόσος* [*nósos*, disease]) is defined as any disease or infection naturally transmissible from vertebrate animals to humans in both rural and urban settings where humans live [1]. The infectious agents involved constitute a diverse range of microbial pathogens such as bacteria, viruses, parasites, fungi, rickettsiae and prions, with a variety of animal vectors or reservoirs, including livestock, companion animals and wildlife [2]. Zoonotic infections result in a wide range of diseases with great socio-economic impact on populations. The most recent example of the worldwide socio-economic impact of zoonosis is the ongoing ‘severe acute respiratory syndrome Coronavirus 2′ (SARS-CoV-2) pandemic. The current respiratory illness responsible for the COVID-19 pandemic is the biggest global challenge since World War II due to the sudden halt in economic activity in both advanced and developing countries, including global poverty that has increased for the first time since 1990 [3]. The reported number of deaths worldwide as of 18 November 2021 is 5.1 million [4]. Based on epidemiological data, the earliest cases of documented SARS-CoV-2 were directly linked to markets selling wild animals in Wuhan (China), without any epidemiological link to other localities, including the Wuhan Institute of Virology (WIV)—the subject of considerable speculation [5]. Although there are precedents for laboratory incidents leading to isolated infections, including SARS-CoV [6], currently, there is no evidence that any early cases of SARS-CoV-2 had any connection to the WIV or any other laboratory. Thus, the most parsimonious explanation for the origin of SARS-CoV-2 is a zoonotic event [5]. Several studies show that bat and pangolin species are a natural reservoir of SARS-CoV-2-like coronaviruses with the potential to infect humans (e.g., [7,8,9]). Although these species are considered to be the hosts from which this fatal pandemic originated, currently, the animal reservoir for SARS-CoV-2 has not been identified.

Animals play an essential role in maintaining infection in nature and contribute in varying degrees to the distribution and actual transmission of infection in human and animal populations. These zoonotic infections have a variety of transmission mechanisms, such as (i) transmission by animal bites or scratches [10,11]; (ii) zoonotic pathogens originating from food animals can reach people through the direct faecal—oral route, contaminated animal food products, improper food handling along the food-processing chain or inadequate cooking [2,12,13,14]; (iii) vectors can transmit zoonotic diseases either actively—by biological vectors, frequently arthropods, such as mosquitoes, ticks, fleas and lice, that may carry pathogens that can multiply within their bodies and be delivered to new hosts, usually by biting—or passively—by mechanical vectors, such as flying insects and other animals such as birds and rats, that can pick up infectious agents on the outside of their bodies and transmit them through physical contact. Even humans, for example, those who are at increased risk of exposure to certain animals, such as livestock farmers and veterinarians, can also unintentionally become carriers of zoonotic pathogens and transmit them to other members of the community [15,16]; and (iv) soil and water resources contaminated with manure may contain a wide variety of zoonotic pathogens, representing an important risk for the transmission of zoonotic diseases [17,18].

## 2. Zoonotic Diseases in Ancient Times: Biological, Archaeological and Literary Evidence

Zoonotic diseases have been in existence for thousands of years and have been hypothesized to have afflicted ancestors of humans from the beginning of the hominin evolution. Food resources were based on the gathering of plants and wild fruits and hunting; therefore, exposure to animal pathogens and cross infection was likely a constant threat to daily life [19]; for example, the analysis of fossilised skeletal remains of hominins has revealed evidence of tuberculosis—likely of bovine origin—[20,21] and chronic brucellosis [22,23]. Numerous lines of archaeological evidence and biological data demonstrate that outbreaks or epidemics of zoonotic infections were most likely experienced around 11,000–9000 BCE in southwest Asia with the ‘First Agriculture Revolution’, when communities of sedentary foragers began to emerge, and the rearing of domesticated livestock developed with the aim of founding a farming economy that produced food rather than collecting it from the wild [24]. The first animals to be domesticated are thought to have been sheep (ca. 11,000 BCE) and goats (ca. 8000 BCE). Both animals were used for their meat, milk and coats and became the main system of food production and for the elaboration of a wide range of secondary products [25,26,27,28,29,30,31,32].

On the other hand, written testimonies sometimes contain mention of human and animal sufferings. The oldest of the veterinary documents is the *Kahun or el-Lahun* papyrus (ca. 2230–1800 BCE), kept in the University College Museum of London. It deals with the diseases of livestock and fish, without reference to human diseases [33,34]. By contrast, the *Ebers* papyrus (ca. 1500 BCE) mentions human diseases [35]. Both documents, corresponding to the Middle Kingdom of Egypt, show that it is one thing to know diseases and another, very different thing, to be aware of the reciprocal animal–human or human–animal contagion. Among other reasons, this was due to their ignorance of pathogen agents in nature, although some hygienic standards were born from simple observation (e.g., rejection of corpses, filth, etc.). As an example, historical references to the bubonic plague are found in the *Iliad* of Homer (ca. 1000 BCE), as well as in the *History of the Peloponnesian War* of Thucydides (430–426 BCE), in the Bible in the *Book of Exodus*, and in the *Metamorphoses* of Ovid (8 CE); references to anthrax are also found in the *Georgics* of Virgil (29 BCE), in *De Medicina* of Celsus (ca. 47 CE), in the *Naturalis Historia* of Pliny the Elder (77 CE) and in *De Differentiis Febrium* of Galen (ca. 200 CE) [36,37,38,39,40].

Today we know enough about several pathogen agents and their host spectrum that we can interpret some endemic situations and epidemics, analysing the ancient written testimonies together with archaeological evidence and biological data. What is indisputable is that the environmental, physical and human factors that affect the onset of disease are numerous and variable in geographical, historical and social terms. In classical Rome or Athens, Constantinople, pre-industrial London and many other examples, there were both predisposing and determining factors that made the urban environment ideal for the emergence of an epidemic or the maintenance of an endemic. In this study, we will examine various predisposing elements typical of the urban environment in relation to zoonotic diseases in the ancient Roman cities of *Pompeii* and *Herculaneum*, both devastated by pyroclastic surges as a consequence of the eruption of Mount Somma–Vesuvius in 79 CE.

## 3. Historical Background of the Roman Cities of *Pompeii* and *Herculaneum*

### 3.1. Geographical Setting

**Figure 1:**
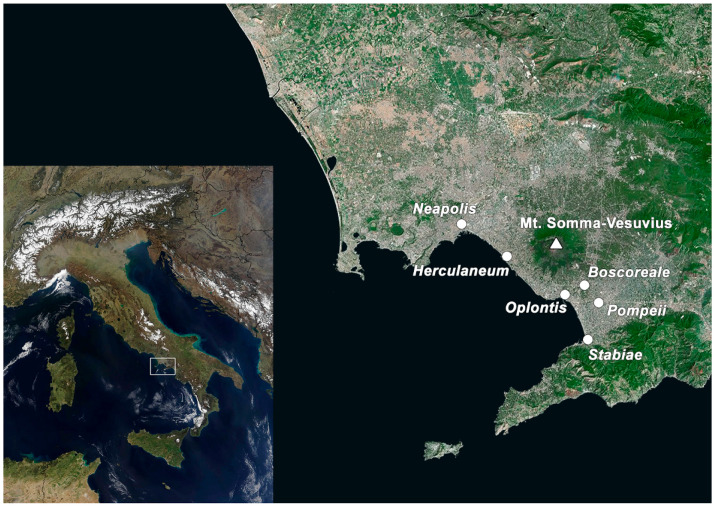
Map showing, with their Latin names, the main cities and towns affected by the eruption of Mount Somma–Vesuvius in 79 CE.

The ancient cities of *Pompeii* and *Herculaneum* were in the Gulf of Naples (region of Campania, southern Italy) (Figure 1). This region was a long volcanic plateau that spread from the Volturno river in the north to the Sarno river in the south, and it was divided into two areas by Mount Somma–Vesuvius. *Pompeii* was on the southeastern side of Mount Somma–Vesuvius, on a volcanic spur up to 40 m above the sea level overlooking the Sarno river. *Herculaneum*, on the western side, spread along the low hillsides of Mount Somma–Vesuvius, which ended in a cliff overlooking the ancient coastline [41,42]. *Pompeii* was a commercial town of strategic importance because it lay on the only route linking the north and south and connected the seaside area with the inland fertile agricultural region. The urban centre (*forum*) was home to the city’s main civic, commercial and religious institutions. Due to its views and moderating maritime influence, *Herculaneum* was considered an ideal resort and residential town, with fewer public and commercial buildings than *Pompeii* [43,44,45].

### 3.2. The Eruption of Mount Somma–Vesuvius in 79 CE

The fame of *Pompeii* and *Herculaneum* is due to the catastrophic volcanic eruption, whose dating was recently re-assessed to October of 79 CE based on new epigraphic testimonies, archaeological finds and volcanological data [46,47,48]. The details of the sequence of relevant disastrous events consequent to the Vesuvian eruption have been handed down to us by Pliny the Younger, the nephew of Pliny the Elder—an important Roman writer and naturalist, as well as the admiral of the Roman fleet. In the form of two letters that he wrote to Tacitus—a Roman historian—to inform him of his uncle’s death, Pliny the Younger gave an eyewitness account of the eruption in a detailed, graphic and objective way. The sequence of events described by Pliny the Younger and the volcanological evidence [49,50,51] has allowed two main phases of the eruption to be established. The first phase was characterised by a widespread dispersal of a cloud of ash, pumice and gases from a high eruptive column, and the second phase was characterised by six pyroclastic surges, which caused extensive destruction in a portion of territories of the Campania region, burying the entire cities of *Pompeii* and *Herculaneum* under tens of metres of pyroclastic material as well as most of the other towns and aristocratic villas surrounding the volcano, such as *Oplontis*, *Stabiae* and *Boscoreale* [42]. In *Pompeii*, the major casualties during the first phase resulted from roof collapses under the weight of the pyroclastic material that rained down during the eruption. Those who abandoned the buildings were killed by suffocation due to the ash-rich atmosphere and harmful gases. On the contrary, in *Herculaneum*, the major casualties were due either to thermal shock or physical trauma as a result of the kinetic energy of the pyroclastic surges [52,53,54].

### 3.3. Investigation and Interpretation of the Different Information Sources

The integral preservation of *Pompeii* and *Herculaneum*, achieved through rapid burial by volcanic deposits tens of metres thick, has made it possible to reconstruct certain aspects of daily life, thanks to the discovery of private houses, public buildings and the skeletal remains of its inhabitants, representing an absolute *unicum* [55]. Buildings are often preserved up to the second floor, as well as wooden furniture, mosaics, frescoes, sculptures and other bronze and iron objects [46,56,57,58,59,60]. Even diverse organic material such as food (e.g., bread, cheese, olives, *garum*, grains, lentils, beans, fresh and dried fruit such as pomegranates, figs, walnuts, almonds) [46,61,62,63] and remains of human faeces [64,65] are well-preserved. Thus, the singularity of the circumstances of burial of these two cities, together with the exceptional nature of the cause of death of its inhabitants and the extraordinary state of preservation of the archaeological and biological material found, has provided researchers with an opportunity, unique in its kind, to reconstruct the life and ways of living of its inhabitants.

In recent decades, the palaeobiological studies carried out on the bone and dental human remains of the victims of the eruption has allowed the obtention of useful information not only to understand the habits and lifestyle of the ancient inhabitants, but also the status of health and diseases with which the general population was affected (e.g., [41,66,67,68,69,70,71,72]). Although bone and dental human remains constitute the primary source through which it is possible to derive direct information on the diseases suffered by the ancient inhabitants, osteological remains from animal assemblages are no less important to provide data that can help to elucidate the close coexistence and animal–human interaction, and the animal-related infections transmitted to humans in the community. Therefore, to provide an in-depth picture of zoonotic diseases of the past, scientific research should not be based exclusively on the human and animal skeletal remains, but the interaction with other data inferable from archaeological evidence is essential, including the urban planning and other biological remains of organic-based nature, but without neglecting information obtainable from literary, epigraphical and artistic sources [73,74].

## 4. Animal–Human–Environment Interaction in the Daily Life at *Pompeii* and *Herculaneum*

### 4.1. Population Density and Housing

At the onset of the Common Era, the Mediterranean region was considered a relatively stable epidemiological pool of diseases to which the population had adapted. However, with its military and commerce expansion and slave trade, the Roman Empire altered this situation through progressive contact with important groups of diseases from neighbouring regions of the Middle and Far East, North Africa and southern and western Europa. Thus, most of the new epidemics that devastated the Roman Empire were introduced by soldiers, merchants, slaves or immigrants [75,76]. In fact, the expansion of the Roman Empire attracted many immigrants to large cities in search of better opportunities, generating several direct health consequences. On the one hand, immigrants constituted an immunologically virgin mass for resident pathogens and those from origins other than their own; on the other hand, they brought their own parasites, vectors and pathogens with them and spread them in their new habitat [75,77,78,79]. In addition, immigration also led to the indirect spread of health-related diseases due to overcrowding and overpopulation in cities, the inevitable lack of hygiene in substandard housings or the systematic non-existence of adequate waste-disposal mechanisms [80,81].

The urban population of *Pompeii* and *Herculaneum*, particularly the wealthy social class, lived in a private single-family residence (*domus*), while those of the less favoured social class lived in tenement block housing with numerous families (*insulae*). In the *insula*, a large part of the population lived in the upper floors over their own shops (*tabernae*)or workshops, in small bedrooms (*cubicula*), in various types of guesthouses (*hospitia*, *stabularia*, *cauponae*, *deversoria*) or simply on the street. The *insulae* accommodated high population densities due to the advantageous use of the housing as a consequence of the reduced interior spaces and a greater number of divisions [82,83,84,85]. They were the cheapest, but the indigents and those who could not afford accommodation when nightfall came—the wages were paid daily—had to resort to other means. Large shanty and slum areas (*tuguria*) were an option [85,86]. The streets of the city were also occupied due to the generous architecture full of arches, vaults, bridges, colonnades, stairs and terraces [82,85,87,88]. Thus, in ancient Roman cities, areas were generated where people lived together in suffocating proximity while garbage, faeces, urine and stagnant water accumulated, along with their associated microfauna (e.g., helminths, protozoa, flies, mosquitoes, fleas, bedbugs, lice and other insects) and their natural hosts (people, domestic animals, rodents, birds) [75,80,81,82]. Table 1 shows the estimated population density for the cities of *Pompeii* and *Herculaneum* compared to other major cities of the Roman Empire (data extracted from [89]).

The distribution and transmission of zoonotic diseases were not only influenced by the housing spaces but also by the different materials used in the construction of the houses. For example, adobe, wattle or heather or cane roofs were good settlements for insects and rodents. Even furniture could host insects and rodents, especially the beds (*lectus cubicularis*). The iconography and archaeological artefacts of *Pompeii* and *Herculaneum* clearly show the main types and uses of the bed during the Roman Empire. In Roman culture, the richest beds had mattresses stuffed with wool or feathers, while the mattresses of the popular classes were stuffed with straw. They generally lacked sheets but had quilts that served to cover them made with very rich textiles, as well as wool or fur blankets [90,91]. In this way, the beds could not only be accessible to insects and rodents, but also the bedclothes could contribute to the biotic load. One example is carbuncle or anthrax, an infection caused by the bacterium *Bacillus anthracis*. It is a zoonosis acquired by livestock (cattle, sheep, goats, horses and pigs), with humans being the accidental host. The main risk of contracting the infection is through contact with infected animals or their products, such as hides, fur or wool [36]. The Latin poet Virgil (Georgics III.478–566; 1st century BCE) cites an epidemic of anthrax that affected the eastern Alps and describes the symptoms in animals: “*[…] then the eyes blaze and the breath is drawn deeply, at times with heavy groans, the depths of the chest strained by long sobs, black blood flows from the nostrils, and the coarse tongue chokes the blocked throat*.” and in humans: “*[…] if anyone handled their hateful clothing, feverish blisters and foul sweat would cover his stinking limbs, and he’d not long to wait before the accursed fire was eating his infected body*” (translated by [92]). Hippocrates, Ovid, Galen and Pliny the Elder also mention anthrax-like epidemics [93,94], although a more “scientific” description is found in De Medicina by the Roman physician Celsus [94].

In the domestic environment, commensal rodents such as the house mouse (*Mus musculus*) live in close contact with humans and domestic animals, where man-made structures provide shelter and abundant food supplies. Thus, the house mouse depends on humans for its food and habitat and cannot survive without human presence [95]. Under commensal conditions, the house mouse is destructive to food supply and human property, undermining and weakening wooden buildings, destroying clothing and gnawing the furniture [96]. In addition to the food they eat, they also contaminate human food supplies with their urine and excrement and can spread various zoonotic pathogens, such as bacteria, viruses and helminths [97,98] through direct transmission (e.g., bite, contact), through ectoparasites (e.g., ticks, fleas, mites and lice) or through endoparasites (e.g., nematodes, cestodes and trematodes) that infest them [99]. Zooarchaeological evidence in *Pompeii* registers a rise in the frequency of house mice and a concomitant decline in the wood mouse (*Apodemus sylvaticus*; see Section 4.3.4. Wild animals), with which the house mouse competes, coincident with the intensification of urbanization in the city [100]. Numerous skeletal remains of *Mus musculus* have been found in both *Pompeii* and *Herculaneum*, indicating a high density of the house mouse in these cities [100,101]. This could be one of the reasons why the houses had domestic animals such as cats to control rodents (see Section 4.3.3. Household animals), which could also be carriers of zoonotic diseases.

### 4.2. Trade Routes

The archaeological finds concerning port structures, together with literary sources, attest that the port of *Pompeii* not only played an important role in the economy of the Campania region—especially for agricultural produce—but also allowed imports to and exports from *Pompeii* even from cities beyond the Empire due to its proximity to the port of *Puteoli*—one of the most important ports of Rome. In addition, *Pompeii* was the entrepôt for the Sarno river valley, which allowed it to trade with other inland cities through fluvial networks. By contrast, *Herculaneum* had a relatively small port with an economy that served local needs [43,44]. In this context, the Roman world represented globalization before this term existed, establishing an extensive trade network, not only maritime and fluvial, but also terrestrial. This allowed animals and their products, as well as people and pathogens, to spread through the cities. The dead spaces between the cargoes carried by carriages and ships allowed animals such as rodents to hide [102]. Rodents are the reservoir for many infectious organisms, which, if transmitted to humans or domestic animal populations, can cause disease outbreaks, often with high morbidity and some mortality.

Together with the house mouse (*Mus musculus*) mentioned above, the black rat (*Rattus rattus*; also known as the roof rat, ship rat or house rat) is also a serious pest in urban and rural environments [103]. Originating from southeast Asia, the black rat arrived in the Mediterranean basin by two different routes: (i) terrestrial or sea-trade with Mesopotamia, where skeletal evidence of black rats was found in Syria in 3500 BCE [104]; and (ii) sea-trade across the Indian Ocean to the Red Sea ports of Egypt, where partially digested skeletal remains were discovered in the stomach of a mummified cat and in mummified birds of prey, some of them dated from Roman times [105,106]. With the expansion of the Roman Empire and the increase in the human population in several cities, especially Rome, large commercial flows were generated that probably facilitated the transport of black rats through the different countries since the intense military and commercial activities of the Roman Empire linked the Near East (Syria, Judea) and the north coast of Africa (Egypt, Cyrenaica) with the western provinces [106]. Although there are a few black rat finds before the Roman times in Europe—the most reliable finds come from southwestern Slovenia and date between 1100 and 800 BCE [107]—the zooarchaeological records increased from the Roman period across Europe [108,109,110,111]. In particular, zooarchaeological finds show that by 200–100 BCE, the black rat was present in *Pompeii* [112,113].

Although the black rats live unprotected in the wild nature of the Mediterranean, they tend to depend on human dwellings and food storage to thrive [102]. Black rat populations not only cause extensive economic damage to crops, stored food, farms, industries and households, but they also harbour and spread diverse zoonotic pathogens, such as viruses (e.g., hantavirus), bacteria (e.g., *Leptospira interrogans*), protozoa (e.g., *Toxoplasma gondii*) and helminths (e.g., *Hymenolepis* spp.) [103]. The dense urbanism of the *insulae*, the baths (*thermae*), the markets (*tabernae*), the shops and workshops, the food houses and the taverns (*thermopolia*, *cauponae*, *popinae*) could favour the establishment of rodent populations so that the distribution of the black rat within Roman cities, including *Pompeii* and *Herculaneum*, could follow a pattern marked by the availability of access to shelter, food and garbage [100,102].

### 4.3. Livestock, Wild, Exotic and Household Animals

One of the most obvious and far-reaching differences between the life of “developed” and mechanized countries of modern times and that of the ancient Roman world lies in the much larger role that animals of all types played in the latter. In fact, there were few aspects of human activity, whether at work or leisure, in which animals were not involved [114].

#### 4.3.1. Exotic Animals

**Figure 2:**
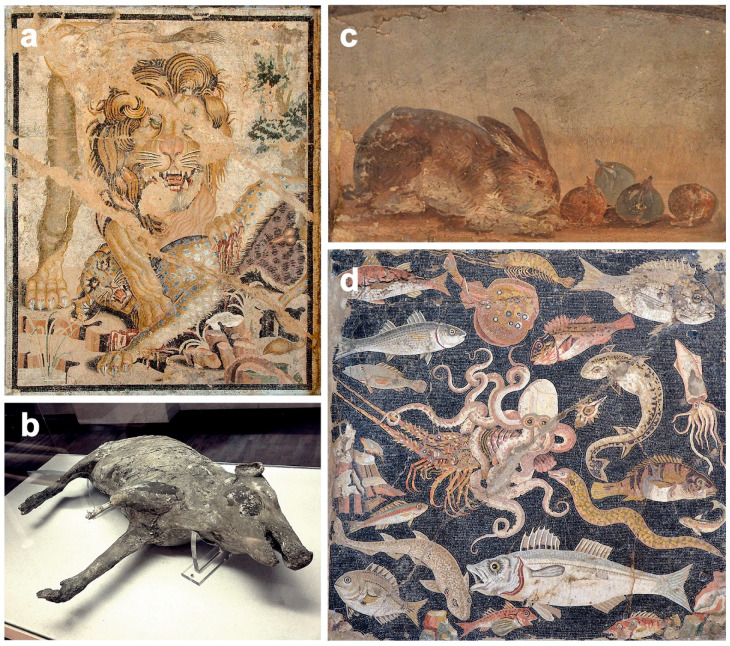
(**a**) Mosaic of a lion attacking a leopard (*House of the Mosaic Doves*, *Pompeii*). Despite the presence of vegetation in this scene, it seems likely that whoever designed this mosaic had observed these animals not in their natural habitat, but in the arena of *Pompeii*’s amphitheatre. Animals were sometimes forced to fight each other after having been starved and tormented to increase their aggression. At other times, animals were part of “hunts” (*venationes*) or pitted against gladiators or criminals condemned to die (*damnatio ad bestias*) [Author: MatthiasKabel. Distributed under a CC BY-SA 2.0 license. Available online: https://cutt.ly/dRCLc99. Accessed: 2 February 2021]. (**b**) Plaster cast of a pig found during the excavation in *Villa Regina* (*Boscoreale*). It certifies the breeding of pigs on Vesuvian farms [Author: RealCarlo. Distributed under a CC BY 2.0 license. Available online: https://cutt.ly/KRCLFFl. Accessed: 30 October 2021]. (**c**) Fresco of a rabbit and four figs (from *Herculaneum*) [Author: Amphipolis. Distributed under a CC BY-SA 2.0 licence. Available online: https://cutt.ly/3RCLM44. Accessed: 30 October 2021]. (**d**) Mosaic of marine life (from *House of the Geometric Mosaics*, *Pompeii*) [Author: Carole Raddato. Distributed under a CC BY-SA 2.0 license. Available online: https://cutt.ly/LRCZwkF. Accessed: 2 February 2021]. All images are of public domain via Wikimedia Commons webpage.

There is no doubt that great mass events were the most popular entertainment event during the imperial era. According to the classical Roman vision of leisure, the great public games (*ludi publici*) were an activity to satisfy the people’s desire for escape and recreation, as well as to guarantee good social order by offering a palliative. Famous is the Juvenal’s expression *panem et circenses* (bread and games) (*Satire* X.77–81; 2nd century CE), with which the poet describes the practice of the *aediles* (the town authorities) of providing free grain and costly public games as a mean to gain political power and to get the consent of the citizens, distracting them from the social problems they were suffering. Of great importance were the *venationes*, as well as the *damnatio ad bestias* shows in which wild and dangerous animals participated, imported to the city expressly for this purpose. *Venationes* were a form of entertainment that involved hunting and killing wild animals, while *damnatio ad bestias* was a particular type of death penalty where those convicted were executed by animals [115,116]. As the Empire expanded, exotic animals such as lions, leopards, tigers, panthers, rhinos, crocodiles, hippos and even elephants began to be incorporated alongside animals of local origin, such as wild boars, foxes, bulls, wolves and bears [117] (Figure 2a). All these animals were captured in their place of origin, and transporting them to the Italian peninsula required covering very long distances by land and sea. According to the Roman writer and statesman Symmachus in his letters (*Epistulae* IV–VI; 5th century BCE), many animals died during transportation, but many others reached the circus and were used as entertainment [114,115,116,118]. Although most of their meat was destined to feed its congeners who waited in the *vivarium* cages for future shows, it could be donated to the public or to the authorities as an act of generosity from the emperor. This gesture was very well received among the attendees, whose purchasing power was not enough to frequently access quality protein. In any case, it was an undoubted potential route of entry for zoonotic pathogens. However, there are no references relating to animals destined for *venationes* and *damnatio ad bestias* shows with different diseases, whether their own or human [81,118].

#### 4.3.2. Livestock

Livestock was among the main resources of Roman society, offering the Mediterranean terrain many animal species with which to raise livestock and obtain good-quality products derived from such [119]. The main sources of information on Roman livestock include ancient Latin treatises, such as those by Cato the Elder (*De Agri Cultura*; 2nd century BCE), Varro (*Res Rusticae*; 1st century BCE), Virgil (*Georgics*; 1st century BCE), Columella (*De Re Rustica*; 1st century CE) and Pliny the Elder (*Naturalis Historia*; 1st century CE). Several artistic representations in mosaics, frescoes and sculptures testify to the implements used in agriculture and livestock and, occasionally, metal parts of these implements have been brought to light during the diverse archaeological excavations. The types of livestock of the ancient Roman world were mainly cattle, equines, sheep, pigs, poultry, rabbits, bees and fish.

Cattle were the most useful animals, producing milk, butter, cheese, meat, fertilizer, horns and bones for tools, leather and fur for the manufacture of clothing [120]. Thus, although cattle breeding was not mainly intended for meat consumption, owning a herd of cattle was a sign of wealth for the Romans. During the winter, a good supply of cereals was reserved for human consumption, for oxen and mules as pack animals to pull ploughs in the field and for animals to transport and mill cereal grains [114,117,120]. Numerous skeletal remains of cows, oxen and mules were found, mainly in the *forum boarium* in *Pompeii* [101,121,122]. There are also numerous artistic representations in mosaics, frescoes and sculptures showing cows, oxen and mules [117].

Horses were also used as a means of transportation, being mostly used for circus races and army *equites* (knights) [114,117,123]. Their breeding was expensive and demanding, so farmers preferred donkeys and mules, which were more resistant and needed less care [123]. Donkeys’ milk was highly appreciated, either for children—especially babies—or for use as a base for cosmetics and therapeutic remedies. In addition, mowers and mills were managed by these animals [117,123]. Various stables have been identified in *Pompeii* (e.g., *House of Amarantus*, *House of Chaste Lovers*, stables belonging to different bakeries) and *Herculaneum* (e.g., a stable in *Insula Orientalis II.1a* belonging to a bakery on the north side of *Vicolo Meridionale*), but their architecture and dimensions are generally not specific enough to assign them, unambiguously, as rooms for oxen, horses or other livestock [114]. Five equine skeletons were found in one of these stables, specifically in the *House of Chaste Lovers* in *Pompeii*, another equid was found in *Herculaneum* [124], and recently three equids were recovered in a large villa in *Civita Giuliana*, a suburb of *Pompeii* [125]. According to classical taxonomic criteria and ancient mtDNA analysis, the classification of the Pompeian equine remains found at the *House of Chaste Lovers* was identified as horses and mules [126,127,128,129], while the Herculanean remains were classified during the excavation phase and later by genetic characterization, as those of a horse [128]. Once again, Roman art provides representations of equines in different artistic works [117].

Sheep were the most widespread livestock because they provided meat for food, wool for clothing, milk for cheese making and fertilizer for cultivating the fields. Goats were also raised, as their milk was highly appreciated, and they also provided leather [117,130]. Numerous bone and dental remains of sheep and goats were found in *Pompeii* and *Herculaneum* [101,121,122], and their representation in artistic works was frequent [117].

Pig farming was widespread, required little care and produced highly appreciated meat [114,122]. Pliny the Elder (*Naturalis Historia*; 1st century CE) collected more than 50 recipes for pork, and similarly, several recipes were reported by Apicius in his gastronomic treatise (*De Re Coquinaria*; 1st century CE). With their meat, they produced long-lasting sausages, with the lard they cooked and with the suet they fed the oil lamps, while with the bristles they made brushes. Numerous bone and dental remains of pigs were found, mainly in the *forum suarium* of *Pompeii* [101,121,122], as well as other skeletal remains uncovered during the excavations of the *House of C. Julius Polybius* in *Pompeii* [131]. Of remarkable beauty is the plaster cast of a pig found in *Villa Regina* in *Boscoreale* (Figure 2b), as well as different artistic works representing this animal [117,131].

Chickens and hens were also very widespread among farmed animals, these being appreciated for their meat, as a supplier of eggs and their manure, which was an excellent corrector of the most sterile vineyards and fields [119,130]; cocks, on the other hand, were also bred as fighting animals and kept for sport [117]. According to Apicius (*De Re Coquinaria*; 1st century CE), ducks and geese were also bred and highly appreciated, especially for their eggs. In addition, bird feathers were used as ornaments for humans and animals such as racehorses [117]. Interestingly, the carbonized remains of a chicken were recovered on the top of an altar as a sacrificial offering in *Pompeii* [46]. As with all other animals, *Pompeii* and *Herculaneum* provide innumerable artistic works depicting these animals [117].

Rabbit and hare farming was appreciated for meat [117] (Figure 2c). According to García [132] (p. 195), on display in the *antiquarium* of *Pompeii* prior to the 1943 WWII bombing, was the skeleton of a rabbit that had been found near the oven of the *Bakery and shop of Sabinus*.

In Roman times, beekeeping was widely practised to obtain wax and honey. The Romans did not have sugar; although they produced a sweet syrup from beets, honey was much more appreciated for its flavour, and it was even used for medical purposes [133]. Many ancient surviving Latin texts on agriculture and natural history provide important information on beekeeping, such as those by Varro (*Res Rusticae* III.16.1–38; 1st century BCE), Virgil (*Georgics* Book IV; 1st century BCE), Columella (*De Re Rustica* IX.2–16; 1st century CE) and Pliny the Elder (*Naturalis Historia* XI.4–1; 1st century CE) [134]. Because Roman hives were made from plant (biodegradable) materials, no material evidence is known. However, five *amphorae* were discovered at *Pompeii* whose inscriptions were used to label them (called *tituli picti*), indicating that they once held honey [133].

Aquaculture was a viable and highly productive industry in the Roman world, focused on raising in *piscinarii* (artificial pools); either in freshwater or marine water, numerous important aquaculture species were raised, such as moray eels, lobsters, sea breams, snappers, sea bass, sturgeons, red mullets, meagres, barbels, pikes, octopus, eels, oysters and mussels, among others [117,135]. According to Martial (*Epigrammata* X.30, 1st century CE), the access to fresh, and even live, fish carried considerable social prestige [136], so with time, incorporating a fishpond into the house or villa became an important status symbol. More than 60 structures plausibly identified as fishponds or tanks have been discovered in gardens and courtyards in *Pompeii* and *Herculaneum* [137]. Numerous remains of fish and seafood from the *Cardo V* sewer were found in *Herculaneum*, showing a high diversity of fish, marine mollusc and marine arthropod taxa [64]. There are also numerous artistic works depicting many fresh and sea fish well attested as being farmed or fished by the ancients, and frescoes and mosaics featuring scenes of seafood or of fishing [137]. Of remarkable beauty are the mosaics from the *House of the Faun*, representing numerous types of fish, and the mosaic of marine life from the *House of the Geometric Mosaics*, both in *Pompeii* (Figure 2d).

**Figure 3:**
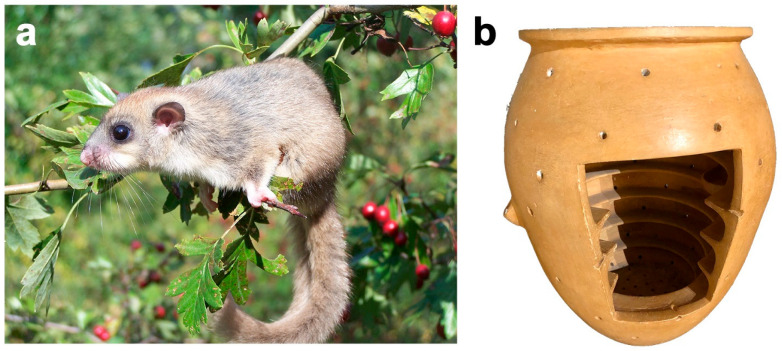
(**a**) Edible dormouse (*Glis glis*), considered a delicacy by the Romans [Author: Azay. Distributed under a CC BY-SA 2.5 license. Available online: https://cutt.ly/bRCLi6u. Accessed: 30 October 2021]. (**b**) Reconstruction of a *vivarium in doliis* (also known as *glirarium*) at the Museum of the Etruscan Academy in Cortona. The *vivarium in doliis* is a terracotta container used for keeping edible dormouse. By inducing hibernation via darkness and confinement, the *vivarium in doliis* would cause the dormouse to fatten [Author: Viscondedeportoseguro. Distributed under a CC BY-SA 4.0 license. Available online: https://cutt.ly/BRCZdek. Accessed: 30 October 2021]. All images are of public domain via Wikimedia Commons webpage.

The large mammals, already mentioned above, were raised mainly as a resource to obtain food. In contrast, small mammals were considered unsuitable for human nutrition, at least among Mediterranean peoples, who generally avoided feeding on rodents and bats, as they were considered harmful to health [138]. However, the edible dormouse or fat dormouse (*Glis glis*) represents an exception from this point of view (Figure 3a). In the context of Roman gastronomy and, in particular, that of the imperial era, the edible dormouse was considered a delicacy [139,140], as confirmed by Martial (*Epigrammata* III.58; 1st century CE), who narrates the unusual peasant tradition of offering edible dormouse along with other products from the rural area as a sign of greeting and respect for the neighbours [138,141], as well as different recipes transmitted by other Latin authors such as Apicius (*De Re Coquinaria* VIII. 9, IX.1.1; 1st century CE) or Petronius (*Satyricon* XXXI.10; 1st century CE). During the II–I centuries BCE, a period in which the Roman world was widely open to foreign influences, especially Greek and Eastern, refined ways of life were overly promoted. This refinement of the society was fought in various ways by a sector of the population that understood that its acceptance and dissemination threatened the ideology and values, those of the traditional Roman, that had made the Roman Republic great [138]. According to Pliny the Elder (*Naturalis Historia* VIII.223, XXXVI.4; 1st century CE), in an attempt to curb the excessive pomp and idleness that prevailed in the social life of patricians and manifested itself in luxury purchases and in the immoderate luxury of banquets, parties and funerals, the most conservative senators promoted the enactment of the so-called *leges Sumptuariae* (Sumptuary laws) that limited the expense that a citizen could incur in various aspects of their day-to-day life. The *lex Aemilia* (78 BCE) stands out, referring to the type and quantity of food that could be served at banquets. Among other things, this law prohibited excessively refined cuisine, as well as the food use of oysters, exotic birds and dormice [119,138,139].

In addition to the historical and literary sources on the alimentary use of the dormouse in Roman times, there are also numerous lines of evidence from archaeological and zooarchaeological studies and research that confirm this practice [100,138,139,140,141]. Thus, even though the dormouse was prohibited for consumption, it was still raised and used as food by the Roman population. Skeletal and dental remains of dormouse have been found in very small quantities, mainly at elite Roman sites, typically rural villages, but also in suburban areas [100,101]. For example, dormouse bones were found in the *forum* of *Pompeii* and in the north courtyard garden of the *Villa of Poppaea* in *Oplontis* [139,140]. However, their recovery, as with all microfauna remains, is highly dependent on effective sieving campaigns during the bone retrieval phase. Although it is possible that the dormice identified in these sites were eaten, there are no cut marks that corroborate this directly [141]. The appreciation that the Romans had for the dormouse in the kitchen is intimately linked to the peculiar breeding techniques in rural villages. As described by Varro (*Res Rusticae* III.15.1–2; 1st century BCE), the dormice were reared and fattened in earthenware containers [140], called *vivaria in doliis* by Pliny the Elder (*Naturalis Historia* VIII.211,224; 1st century CE) [141] (Figure 3b). The internal surface of these *dolia* was characterized by the presence of parallel and concentric protrusions—called *semitae*—or by a single continuous helical protrusion through which the animals could move without getting dirty with their own faeces that accumulated at the bottom of the container. The surface of these *vivaria in doliis* (also known as *gliraria*) had many small holes, presumably to aerate the container. In addition, the openings connected to small containers would probably allow the animals to be provided with drinking water and/or food [119,140]. Several *gliraria* have been found, but only nine can, with reasonable certainty, be identified as such. Although some of them were found in different Roman provinces, six were found around the area of Mt. Somma–Vesuvius: four in *Pompeii*, one in *Herculaneum* and one in *Boscoreale*. The fact that *gliraria* were mainly found in *villae* suggests that the consumers of dormouse were predominately members of the Roman elite [140].

#### 4.3.3. Household Animals

The Romans benefited from collaboration and companionship with different animals, and both then and now, the dog was their favourite companion animal [142]. According to Virgil (*Georgics* III.404–413; 1st century CE), Columella (*De Re Rustica* VII.11.1–2; 1st century CE), Strabo (*Geographica* XV.1; 1st century CE), Grattius (*Cynegetica* 179; 1st century CE) and Pliny the Elder (*Naturalis Historia* VIII.61; 1st century CE), in ancient Rome, the dog performed various functions [117,143,144,145]. On the one hand, it was used in popular shows and in the largest circuses, where they fought against each other or against aggressive bears, tigers and lions [117]. For this, the *molossus* was used, a canid with a strong complexion endowed with piercing fangs and a strong jaw, as described by Virgil (*Georgics* III.404–413; 1st century CE) and Varro (*Rerum Rusticarum* II.9; 1st century BCE). Seeing its potential in shows, the *molossus* was also introduced in warlike contexts, constituting the so-called *canes pugnaces* (fighting dogs). They were brought to the battlefield in packs of several dozen by special units of the Roman legion [143].

On the other hand, dogs were also highly prized on hunts. The Romans called hunting dogs *canes venatici* (sporting dogs) and divided them according to the kind of hunting for which they were predestined: the *sagaces* were those used to follow the tracks of the prey (hounds); the *celeres* were the fast dogs used for the pursuit of the prey, with a special preference for the greyhound (*canes vertragus*); and the already commented *canes pugnaces*, used to attack prey, especially wild boars and other wild animals. The rural environment was another place where dogs stood out, also being used for herding or guarding the villages. The *canes pastorales* (shepherd dogs) were used for the care and transport of livestock. In addition, they defended livestock from predators that lurked on the roads and in the fields and forests. The *canes villatici* (watchdogs) were the dogs destined for the custody of *domus*, *insulae*, *villae* or workshops, warning if strangers appeared [143,146].

**Figure 4:**
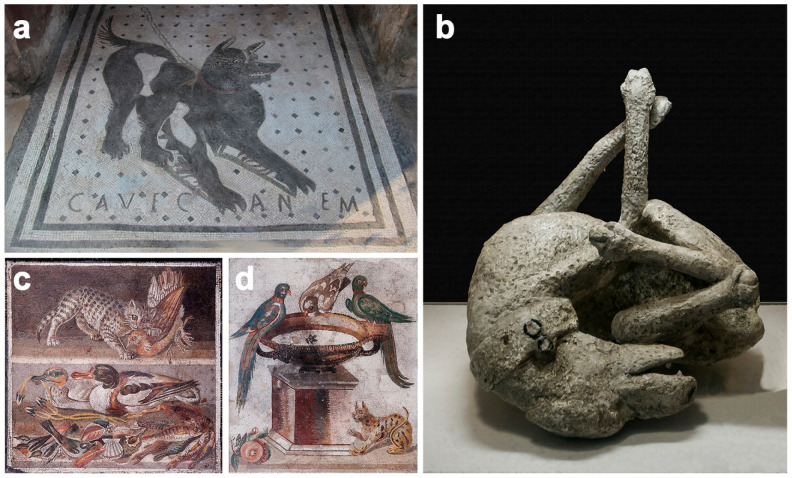
(**a**) Mosaic at the entrance door to the *House of the Tragic Poet* (*Pompeii*) depicting a dog served as a warning to intruders, with the inscription ‘*Cave canem*’ (Beware of the dog!) [Author: Sailko. Distributed under a CC BY-SA 4.0 license. Available online: https://cutt.ly/JRCZnid. Accessed: 30 October 2021]. (**b**) Plaster cast of a watchdog found in the *House of Vesonius Primus* (*Pompeii*), showing his leather collar with the two bronze rings for the chain [Author: Jebulon. Distributed under a CC0 1.0 license. Available online: https://cutt.ly/TRCZmFG. Accessed: 30 January 2021]. (**c**) Mosaic from the *House of the Faun* (*Pompeii*) representing a cat attacking a partridge [Author: Marie-Lan Nguyen. Distributed under a CC-PD-Mark 1.0 license. Available online: https://cutt.ly/ARCZQYm. Accessed: 30 January 2021]. (**d**) Mosaic from the *House of the Faun* (*Pompeii*) depicting parrots, a dove and a cat [Author: Marie-Lan Nguyen. Distributed under a CC-PD-Mark 1.0 license. Available online: https://cutt.ly/wRCZWLt. Accessed: 30 January 2021]. All images are of public domain via Wikimedia Commons webpage.

At the end of the Republican period (during the 1st century BCE), the keeping of dogs to serve as guardians of the home became fashionable among the wealthier Roman classes. Thus, in some Roman *domus*, we find represented in mosaics how these ‘companion’ dogs served as a warning to intruders. They were represented with the inscription ‘*Cave canem*’ (Beware of the dog!). As an example, we can highlight the mosaic at the entrance door to the *House of the Tragic Poet* (Figure 4a) or the mosaic in the foyer of the *House of Paquius Proculus*, both in *Pompeii* [117]. It was common for these mosaics to represent a *molossus*, a dog whose impressive musculature impressed and intimidated anyone entering the *domus*. The plebeians, generally with scarce economic resources, could not afford this type of guardian animal, so they had geese instead. Their territorial character, along with their loud squawk, made them an excellent and inexpensive guardian.

In a society as refined as the Roman one, the dog also became a much-loved companion. To serve as pets, it was enough to be docile and faithful, like the small *canis catelli*. Their sole purpose was to be an entertainment for children, a flea and fly deterrent for its owners and a symbol of social status for wealthy women. This class of dogs (lapdogs) were so appreciated that the description that the Roman poet Martial (*Epigrammata* I.109; 1st century CE) makes of the dog named Issa of his friend Publio reflects it clearly [147]. Martial says: “*[…] Issa is more pure than the kiss of a dove. Issa is more loving than any maiden. Issa is dearer than Indian gems. […] That her last hour may not carry her off wholly, Publius has her limned in a picture, in which you will see an Issa so like, that not even herself is so like herself.*” (translated by [148]). However, these pet dogs were not common among the plebeians because, generally, having a dog that did not work—either as a guardian, shepherd or hunter—was beyond most people’s comprehension.

Numerous ancient Latin texts and artistic representations in mosaics, frescoes bas-reliefs, statues and other archaeological artefacts evidence the presence and diffusion of those types of dogs [117]; for example, the mosaics depicting watchdogs found in the *Houses of Paquius Proculus*, *Vesonius Primus* and the *House of the Tragic Poet* in *Pompeii*; the mosaics (*House of the Wild Boar*), frescoes (*House of the Gladiators*, *House of the Wild Boar*, *House of Menander*) and statues (*Domus of L. Rapinasi Optati, House of the Deers*) depicting hunting scenes in *Pompeii and Herculaneum;* and archaeological remains of what are considered doghouses in the *House of Achilles* and the *House of the Garden of Hercules* in *Pompeii*. Furthermore, numerous skeletal remains of dogs have been found in *Pompeii*, such as, for example, those found in the *House of Amarantus*, *House of Paquius Proculus*, *House of Romulus and Remus* and *Villa of Diomedes* in *Pompeii*. Interestingly a plaster cast of a watchdog was found in the *House of Vesonius Primus* in *Pompeii*, showing his leather collar with the two bronze rings for the chain (Figure 4b).

The cat is another animal found in the Roman domestic environment. There is some evidence of coexistence between cats and men as early as ancient Egypt—where they were traditionally venerated as a sacred animal—and later also in Greece, although there this animal never gained the prestige it had in Egypt [114,149]. It was in the 1st century CE when the cat would begin to be known in the Roman world. The Romans used colubers and mustelids, such as weasels, stone martens and ferrets, for the control of rodents [142,149]; but they soon realized that cats were more easily tamed and appreciated the services both as a working animal—to eradicate rodents from their homes—and as a companion animal. Therefore, according to Pliny the Elder (*Naturalis Historia* X.73; 1st century CE), domestic cats replaced mustelids at the beginning of the Christian era [150]. During the conquest campaigns, the Romans took cats with them, contributing to its spread throughout Europe [149]. The reason they took them was so that they would hunt the many rodents in the camps and winter quarters. In addition, after passing through Egypt, due to its link with Isis, the cat was considered an animal that symbolized victory. Traces of the presence of the cat have been found in all the regions conquered by the Romans [105,149,150].

Although the skeletal remains of many diverse animals abounded in *Pompeii* and *Herculaneum*, the skeletal remains of cats are extremely rare. The skeletal remains of a cat have only been found in a large commercial vineyard at *Pompeii* [151] and in the *forum* (cited in [101]). However, in *Pompeii*, there is no shortage of representations of the feline: of remarkable beauty, for example, is the mosaic from the *House of the Faun* that depicts a cat attacking a partridge, now kept in the Naples National Archaeological Museum (Figure 4c). The same museum exhibits another Pompeian mosaic depicting parrots, a dove and a cat, also from the *House of the Faun* [117] (Figure 4d). In any case, it should not be forgotten that *Pompeii* had many contacts with Greece and Egypt; that is why it is believed that the ‘Pompeian’ cats, the few that there were, managed to save themselves at the first signs of the catastrophe [117]. However, these are just mere speculation.

#### 4.3.4. Wild Animals

The presence of loose animals in the city was a constant and was part of the ancient Roman urban landscape. Dogs were abundant even in the homes of the poorest people, not only as guardians of modest homes, but also used to dispose of food scraps (as pigs had also been used) [114]. Tanners, for their part, hunted them to make leather, and the most disadvantaged people probably consumed meat from stray dogs as a source of protein in the absence of other prey, this being an atypical way of acquiring hydatid cysts [85].

However, there were many other animals that roamed the public roads of Roman cities. Horace (*Epistulae* II.2. 72–75; 1st century BCE) describes the city of Rome as noisy and chaotic, emphasizing how busy the streets were and the lack of order when people and animals made their way down the street together. As an example, Horace mentions a muddy sow walking calmly through the city [152]. At that time, there were no slaughterhouses as we know them today, but the animals were bought in the corresponding livestock market (*forum boarium*: cattle market; *forum suarium*: pig market) and driven alive, urinating and defecating in the streets, to the *macellum* (public market dedicated to the sale of meat and fish market) and specialized butchers’ shops, where they were slaughtered, disembowelled, dismembered and sold. Undoubtedly, the continuous traffic of animals such as pigs, sheep and cattle contributed to the general dirtying of the streets with excrement. In addition, leftovers were often dumped directly onto the street, which, together with the low levels of sanitation and poor standards of public health, implied a high risk of numerous diseases such as rabies, bovine tuberculosis, parasitosis (e.g., scabies, echinococcosis, trichinellosis, taeniasis), ringworm, brucellosis, leptospirosis and many more. If the frequent faecal contamination of water and food is also included, this list would be extended to other diseases, such as cholera, hepatitis, dysentery, typhoid, salmonellosis, colibacillosis, etc. [85,114].

As for rodents, the black rat (*Rattus rattus*) would be the protagonist, already mentioned above (see Section 4.2. Trade routes). However, we can also highlight the wood mouse (*Apodemus sylvaticus*). The wood mouse, like the house mouse (*Mus musculus*) (see Section 4.1. Population density and housing), is an agricultural pest that damages cereals, orchards, pastures, horticultures and tubers [95,153]. However, unlike the house mouse, the wood mouse can survive in environments where humans are not present, allowing it to colonize a wider range of environments and travel between human and wild environments. Thus, this higher level of mobility could have influenced a greater spread of diseases. The spread of diseases transmitted by rodents is not only influenced by available food and environmental conditions, including pathogens and disease vectors present in the environment, but also by factors specific to the species itself, such as the population density, the size of the distribution area, and the variety of environments that the species is capable of inhabiting [154]. Thus, wood mice are generally more dangerous vectors of diseases than house mice. Their greater range and greater flexibility to the habitat in which they live provide them with greater opportunities to contract diseases or contract disease-bearing parasites and a greater ability to transmit these diseases and parasites over long distances. A contemporary example would be the transmission of the pulmonary syndrome by the hantavirus, a dangerous respiratory infection that is transmitted indoors when wild rodents invade them in bad weather situations [155].

House mice frequently outnumber wild mice, suggesting that a high density of house mice in an ancient city may have had the additional effect of protecting the human population from the importation of external diseases by wild mice [100]. If *Pompeii* and *Herculaneum* had not been occupied by a high density of house mice (*Mus musculus*), as shown by the amount of skeletal remains found in these cities, they would probably have been occupied by wood mice (*Apodemus sylvaticus*), as suggested by the evidence of the different excavations carried out [100].

Summarizing, animals—whether livestock, wild, exotic or household animals—accompanied the Romans in most of their daily chores, constituting a health risk for people in close contact with them. Thus, bites, scratches and allergies would be the most common threats and would result in localized infections; however, there are other infections—parasitic, bacterial, viral and fungal—that could be transmitted by direct contact with these animals, their urine and excrement or through arthropods, such as dogs (e.g., rabies, hydatidosis, toxocariosis, leptospirosis, asthma), cats (e.g., toxoplasmosis, toxocariosis, cat scratch, rabies, pasteurella), rodents (e.g., hantavirus, leptospirosis, salmonellosis, leishmaniasis, toxoplasmosis), pigs (e.g., cysticercosis, taeniasis, balantidiasis) or birds (e.g., psittacosis, cryptococcosis, histoplasmosis, avian flu), among others (e.g., [98,99,114,156,157]).

### 4.4. Occupational and Work-Related Zoonotic Diseases

After the Social War (91–87 BCE), *Pompeii* was elevated to the status of Roman *colony* under the name of *Colonia Cornelia Veneria Pompeianorum* (80 BCE), whereas *Herculaneum* received the lower rank of *municipium* (89 BCE) [158]. This situation led to an enormous development of both cities, both from an urban and economic point of view. There was a great commercial boost thanks to the fertility of the volcanic soil and its excellent geographical position in the centre of the Gulf of Naples. Thus, commerce played a fundamental role in the local economy, as evidenced by the abundance of shops and taverns unearthed in *Pompeii* and *Herculaneum*. Mainly agricultural products were commercialized, although artisanal or “industrial” activities related to the transformation of farm products were also developed [43,44].

The historical sources, particularly from the I–II centuries CE, bear witness to the great variety of commercial activities, occupations and trades in these ancient Roman cities [44]. Economic activities of all kinds were located throughout the city, especially along the main arteries of both cities: the *Via dell’Abbondanza* in *Pompeii* and the *Decumanus Maximus* in *Herculaneum*. Among all the commercial activities, some of them represented certain risks, with some specific activities and trades that increased the probability of zoonotic infection. These zoonotic occupational diseases are common when there is close contact at work with animals or animal products. The main occupations with a risk of zoonosis were those in which workers were in contact with: (i) infected animals; (ii) materials or products from infected animals; (iii) secretions from infected animals, including saliva, blood and faeces; (iv) aerosols or powders contaminated with secretions from infected animals; (v) soil or water contaminated with materials or secretions from infected animals; or (vi) infected vectors implicated in the transmission of a zoonosis [159,160]. The occupations with the highest risk of zoonotic infections were those of the agricultural industry, as farmers had close contact with potentially infected animals during land cultivation activities. Other hazardous occupations with close contact with animals included those involved in animal husbandry (e.g., aviaries), those involved in the animal trade (e.g., importing exotic animals for public games), activities of handling and processing of potentially infected animal products, as well as those activities related to the cleaning and hygiene of the facilities where animals or animal products were kept. Certain zoonoses are particularly associated with contaminated water, so the risk of infection was high in occupations that were in close contact with water, including certain occupations related to wastewater treatment.

#### 4.4.1. Agriculture

Agriculture was the basis of the Roman economy, whose main concern was feeding the vast number of citizens and legionaries who populated the Mediterranean region [44,161]. However, with regard to excess human waste, growing cities such as *Pompeii* and *Herculaneum* began to have problems. This situation encouraged the Romans to find other ways not only to dispose of this progressively accumulating waste, but also to use them for commercial exploitation [162].

Human urine was not only used for mordanting certain dyestuffs and for cleaning purposes [85,163] (see Section 4.4.3. Textile industry: Laundries (*fullonicae*) for more details), but also, because it contains significant levels of nitrogen, phosphorus and potassium, it met the requirements for an efficient fertilizer for crop cultivation [164]. For example, the ancient Roman writer expert in agriculture Columella (*De Re Rustica* II.15, 1st century CE) asserts that “*[The] human urine, which you have let grow old for six months, is fitter for shoots and young trees. If you apply it to vines, or to apple-bearing trees, there is nothing that contributes more to make them bear abundance of fruit; nor does this thing only produce a greater increase, but it also improves both the taste and the flavour of the wine, and of the apples.*” (translated by [165]). Columella (*De Re Rustica* II.23, 1st century CE) also suggests watering pomegranate trees with human urine, making them juicier and tastier [166].

Another commercial exploitation involving human waste was the use of excrement as fertilizer. In this regard, in the Roman world, human excrement was used as a complementary or substitute of animal manure to fertilize the soil of croplands [162]. Columella (*De Re Rustica* I.6.24, 1st century CE) promoted the use of mixed animal and human excrement (*stercorata*) since he noticed that manure from both barnyard and *thermae* (baths) worked best [167]. The Romans believed that nature did not admit waste and included everything natural in a spiral of life and death. In this way, it can be said that the Romans had realized that recycling the bodily waste of humans and animals was a good thing for the productivity of the soil [167]. Cato the Elder (*De Agri Cultura* LXI, 2nd century BCE) confirms the importance of manure to nourish croplands: “*What is the first principle of good agriculture? To plough well. What is the second? To plough again; and the third is to manure […]*” (translated by [168]). Other Latin author experts in agriculture, such as Varro and Columella, attest to the practice of using human excrement to manure the croplands. Varro (*Res Rusticae* I.38.1–3; 1st century BCE) informs that “*[…] several kinds [of manure] have different qualities.*”, and states that “*[…] the best manure is that of birds, but the best of all is […] the manure of pigeons because it is the hottest and causes the land to ferment. […] The manure next in value to that of doves is human faeces, and third that of goats and sheep and asses. The manure of horses is of the least value on corn land […].*” (translated by [168]). On his part, Columella (*De Re Rustica* II.15; 1st century CE) asserts that “*There are three principal sorts of dung:* that *produced by fowls,* that *by men, and* that *by cattle. Of fowls that [which] is reckoned the best, which is brought out of pigeon-houses. […] The second is that which men make, if it be mixed with the other filth and sweepings of the manor-house; for it is naturally very hot by itself, and therefore burns up the ground.*” (translated by [165]).

The literary sources verify that the Romans used animal and human waste in agriculture, but apparently, human waste was not the first choice, despite its copious availability in urban centres (see Section 4.5. Latrines, sewers and baths (*thermae*) for more details), but it was frequently used as a supplement to animal manures [85,167]. It is easy to understand how the use and management of human faeces as fertilizer could have constituted a danger to public health as these, not being subjected to any specific disinfectant treatment, could have kept various pathogens within them which, once dissolved, would infiltrate the soil and aquifers and then consequently contaminate the foods through which it was easy for the population to become reinfected [169,170]. Edible vegetables and fruits fertilized with human waste held considerable risks and could be contaminated with various viruses, bacteria and worm eggs. Moreover, if disease carriers such as insects, rodents and pets came into contact with excrement, especially human excrement, and then came near food or drinking water, the problem could become chronic [85,167]. Scobie [85] discusses the various diseases to which Romans were susceptible in the light of their usage of human excrement as fertilizer (e.g., cholera, dysentery, gastroenteritis, infectious hepatitis, leptospirosis, typhoid fever) and lists innumerable common genera of pathogenic organisms that can be found in water contaminated by infected faeces (e.g., salmonellosis, colibacillosis).

#### 4.4.2. Livestock Farming

As already mentioned above (see Section 4.3.2. Livestock), livestock farming and pastoralism were among the main economic resources of Roman cities, being related to a series of activities in which livestock were handled. Within these activities, the operations that would produce a transmission of zoonotic diseases would be, for example, (i) assistance to the birthing of animals; (ii) care of the offspring; (iii) feeding of livestock; (iv) transportation of livestock; (v) milking of dairy breeds (i.e., goats, sheep, cows); (vi) sheep shearing; (vii) hooves’ care; (viii) amputation of bovine antlers; (ix) caring for sick animals; and (x) cleaning of animals and their facilities [171]. Often, during these activities, Roman workers had to stay with the animals or even grab them, which would represent a series of risks that would materialize in the form of accidents whose origin would be found in the unpredictable reaction that the animals could have (e.g., scratches, bites).

#### 4.4.3. Textile Industry

After agriculture, one of the most important artisanal economic activities was the clothing industry, a large, highly profitable and highly organized trade in different sectors [172].

**Tanneries (*officina coriariorum*).** The tanning industry was the industrial sector that produced hides and skins by recovering and enhancing a by-product of the food industry: raw animal skin from slaughter. The *officina coriariorum of M. Vesonius Primus* is the only tanning complex identified in *Pompeii,* where the rawhides were macerated and processed to be transformed into leather. The tannery was operational in 79 CE and was divided into separate compartments necessary for all stages of the tanning process [173]: in the courtyard, there was a stone bench for skinning the animals, and the courtyard was also used for the storage of fresh leathers to be treated; the tanning of fresh leathers was carried out in a complex of tanks arranged under a portico; under the arcades of the courtyard there were the systems for tanning leather, finishing, cutting and making leather objects. Furthermore, in this tanning complex were found tools characteristic of leather processing, suitable for each of the stages of tanning: a semi-circular blade knife used to epilate and deflesh the hides, a tool with a rectangular metal blade to soften and smooth the leather, and a circular blade knife used by tanners and shoemakers to pre-cut pieces of leather [173,174]. In ancient *Pompeii*, the tanning industry was closely linked to that of wool; in fact, *M. Vesonius Primus* was not only the manager of a tannery but also of a *fullonica* (industry for cleaning clothes) among the many that were present in the rich Vesuvian town (see Section 4.4.3. Textile industry: Laundries (*fullonicae*) for more details).

Tannery workers were at considerable risk of developing occupational contact dermatitis, and this inflammation of the skin could constitute the main danger to health caused by contact of the skin and certain chemicals and tools used in the handling and elaboration of raw hides and skins [175]. Other health risks could be carbuncle or anthrax, an infection caused by the bacterium *Bacillus anthracis*, the main risk being contact with skin or hair, bone products and wool or with infected animals. Inhalation of anthrax could occur if anthrax spores entered the lungs through the respiratory tract. This type of infection can be contracted when workers inhale airborne anthrax spores during processes such as leather tanning and wool processing [40,176].

**Figure 5:**
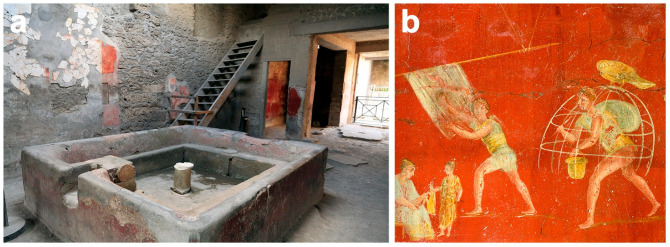
(**a**) *Fullonica of Stephanus* (*Pompeii*), showing the atrium with laundry basin [Author: WolfgangRieger. Distributed under a CC BY-SA 4.0 license. Available online: https://cutt.ly/xRCZOId. Accessed: 30 October 2021]. (**b**) Roman fresco from the *Fullonica of Lucius Veranius Hypsaeus* (*Pompeii*). The man on the left is busy brushing wool cloth. The man on the right, standing beneath a caged dome, is engaged in fabric whitening via sulphurized fumigation. An owl is perched on top of the cage, likely a symbol of Athena, protector of the *lanaiuoli* (i.e., companies of wool merchants) [Author: WolfgangRieger. Distributed under a CC-PD-Mark 1.0 license. Available online: https://cutt.ly/DRCZPWP. Accessed: 30 October 2021]. All images are of public domain via Wikimedia Commons webpage.

**Laundries (*fullonicae*).** Theoretically, five types of textile workshops can be distinguished: (i) initially raw wool was washed and combed in the *officinae lanifricariae;* (ii) then the wool was dyed in the *officinae tinctoriae*; (iii) spinning and weaving were carried out in the *textrinae*; (iv) the wool was felted in the *officinae coactiliariae*; and (v) the *fullonicae* were the establishments where the finishing of woollen clothes took place, but which also took on the functions of a laundry service [177]. However, this distinction, based on the archaeological record, often does not allow a clear differentiation between the different types and all of them are named with the general term *fullonicae* [178] (Figure 5a).

The *Fullonica of Lucius Veranius Hypsaeus* in *Pompeii* exemplifies the arrangement and furnishing of a Pompeian *fullones*’ establishment, in which depictions of a textile press and work scenes were found in the inner courtyard (Figure 5b). These iconographic representations give us important information about the production sequence. The first operation was that of washing, which was done with “fuller’s earth”—a type of clay mixed with natural alkaline chemicals such as carbonate of soda, potash and human urine—to remove any oily material. This was done in tubs filled with a cleaning solution and cleaned by the *fullones* (laundry workers) treading upon them. When dry, the cloth was brushed and carded to raise the nap to make the surface even. The cloth was then fumigated with sulphur and bleached in the sun while spread out over a frame. Cleaning soiled clothes was another function of the *fullones* [58,172]. Science tells us that bacteria transform the urea present in urine into ammonia [179]. The Roman poet Catullus (*Carmina* 39.20, 1st century BCE) could not have known this, but he certainly knew the whitening power of urine on the teeth, of which he expressly speaks in one of his *Carmina*, a sign that it was a practice adopted by his contemporaries [180]. However, ammonia has degreasing properties and fixes natural colours, so human urine was also used as a detergent, as a mordant for some dyes and as a bleach in the textile industry [173].

The collection and use of urine by *fullones* for mordanting certain dyestuffs reveal an area of private enterprise in the disposal and commercial exploitation of human waste in Roman cities [85]. Thirty-nine *fullonicae* were active in *Pompeii*, for which large quantities of urine were indispensable. That urine was widely used at that time is demonstrated by the story of the famous *vectigal urinae* (the urine tax), which tanners and *fullones* were forced to pay on the urine collected in public latrines. It was imposed by Emperor Vespasian, aware of the vast use of urine in the textile industry. The Roman historian Suetonius (*De vita Caesarum*, Vespasian 23.3, 2nd century CE) reports that when Vespasian’s son Titus complained about the disgusting nature of the tax, his father held up a gold coin and asked whether he felt offended by its smell (*sciscitans num odore offenderetur*). When Titus said “No”, Vespasian replied, “Yet it comes from urine” (*Atqui ex lotio est*) [179].

As can be imagined, being a *fullo* was certainly not a good job, and even living near these commercial buildings was not supposed to be pleasant. To get the urine needed, the *fullonicae* encouraged pedestrians to supply urine through jars hung on the wall placed in front of these shops [162,181]. References to the olfactory nuisance caused by fulling are few and fragmentary. Martial (*Epigrammata* VI.93.1–2) associates *fullonicae* with bad smells in an epigram, whose text invokes a situation in which one of the overused *dolia curta* (urine container) (see Section 4.5. Latrines, sewers and baths (*thermae*) for more details) was accidentally broken, either knocked over in the street or during transport by the *fullones* and the odour circulated freely through the air [182]. In any case, since the cloths at the end of the procedure were clean and odourless, the practice of recycling urine and its industrial use continued for centuries until chemistry was able to synthesize urea and produce synthetic detergents. Detailed information on the health implications because of the handling of human faeces and urine is shown in Section 4.5. Latrines, sewers and baths (*thermae*).

#### 4.4.4. Storage and Grain Processing, and Bakeries (*Pistrina*)

Ancient literary sources (e.g., Cato de Elder *De Agri Cultura*, 2nd century BCE; Varro *Res Rusticae* Book III, 1st century BCE; Columella *De Re Rustica*, 1st century CE; Pliny the Elder *Naturalis Historia*, 1st century CE; Apicius *De Re Coquinaria*, 1st century CE), as well as archaeological and biological evidence, show that legumes (e.g., faba bean, lablab bean, cowpea, lentil, chick pea, common pea, grass pea, white lupine, bitter vetch, fenugreek, mung bean, alfalfa), together with cereals (e.g., wheat, barley, sorghum, durum, einkorn, emmer, millet, oats, rice, rye), constituted most of the nutritional and energy needs of an individual in ancient times [183,184,185,186]. As the Roman Empire expanded, huge amounts of food were needed to supply the soldiers, and urban residents with little or no production of their own crops depended on large amounts of imported food supplies. This situation meant not only more intensive agriculture in rural areas, but also an increasing importance of markets and large-scale trade throughout the Mediterranean, with the frequent arrival at Roman ports of cargoes from the islands of Sicily and Sardinia, North Africa and Egypt [161,187,188].

To have cereals and legumes available throughout the year, it was necessary to store them properly in specially designed structures, known as *horrea*, with a view to their long-term conservation [189]. Latin authors such as Varro (*Res Rusticae*, 1st century BCE) and Pliny the Elder (*Naturalis Historia*, 1st century CE) described different storage practices, stating that the storage of raw cereals and legumes in a sealed, cool and dry environment would prevent germination and deterioration, allowing long-term storage. Under conditions of high humidity and temperature, contamination and deterioration of the entire supply would be encouraged, as well as rodent and insect infestations and mould growth [190,191,192]. Rodents such as the wood mouse (*Apodemus sylvaticus*) (see Section 4.3.4. Wild animals) were agricultural pests that damaged grain and seed stores [95,153]. Insects, such as worms, weevils and beetles, could lay their eggs before cereals and legumes were harvested, so larvae could emerge, feed on stored supplies, and contaminate them during storage [193]. Mould could develop under ideal humidity and temperature conditions, contaminating supplies by itself and attracting other insects that feed on the mould itself.

The *horrea* were not the only structures in Roman cities that stored, protected and distributed cereals, legumes and other foods. Small outlets, such as bars, shops and bakeries, represented important urban establishments where residents could buy these foods or products made from them on a regular basis [194,195,196]. In cities such as *Pompeii* and *Herculaneum*, various shops sold cereals, legumes, nuts and other foods [197]. To preserve these food products, Roman shops and bars featured distinctive architectural elements, such as large earthenware jars (*dolia*) embedded in a masonry service counter [194,195,196], which provided them with great advantages. The ceramic material in these jars was an effective thermal insulator capable of providing stable levels of temperature and humidity for the contents. In addition, being enclosed in concrete counters, additional insulation was provided to these containers. Thus, properly sealed, these jars protected their contents from rodents, insects and other pests that could otherwise infiltrate. Therefore, this masonry retail service counter worked in stores as a refrigerator for the preservation of food, especially cereals and legumes, in stable, cool and dry environments [190].

**Figure 6:**
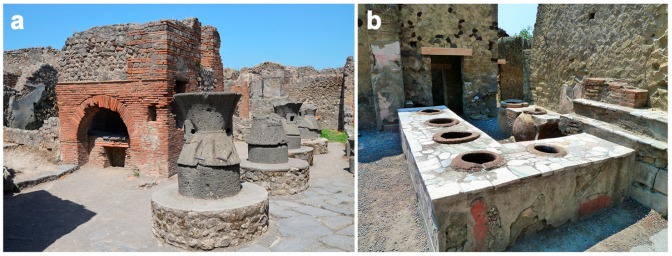
(**a**) The remains of the *Pistrinum of Popidius Priscus* (*Pompeii*) is a fine example of a bakery in which the whole cycle of breadmaking from milling to baking the bread was performed. The *pistrinum* contains four large millstones made from porous lava, traces of a stable, four storage rooms and a large oven, which was used for baking the bread [Author: Carole Raddato. Distributed under a CC BY-SA 2.0 license. Available online: https://cutt.ly/XRCZVTW. Accessed: 11 August 2021. (**b**) *Thermopolium* in *Herculaneum*, a commercial establishment where it was possible to purchase ready-to-eat food [Author: Carole Raddato. Distributed under a CC BY-SA 2.0 license. Available online: https://cutt.ly/FRCZMgT. Accessed: 30 October 2021]. All images are of public domain via Wikimedia Commons webpage.

Other structures of considerable interest are those reserved for the elaboration of certain foods. The kitchens of most Roman houses did not have sufficient facilities for making bread, so it was bought in the *pistrinum* (bakery). The *pistor* (baker) and his assistants were in charge of the entire production process in the same establishment, which had various specific rooms for each task: (i) storage: textile sacks of cereals were stored in the attic of the establishment; (ii) grinding: from the attic, the sacks were poured onto the *catillus*, the upper concave and hour-glass stone of the mill that rotates over the *meta*, the stationary lower cone-shaped stone, both made of strong volcanic rock. Both pieces formed a grinding wheel that was turned by animals or by humans—usually slaves—from which the flour was produced. The animal-driven rotary mill significantly increased the flour output and quality as equids (i.e., horses, donkeys or mules) could drive the mill for hours at a time, saving the humans from the drudgery of milling; (iii) kneading: in another area of the establishment there were kneading benches; (iv) baking: once the bread was shaped, it was passed to the *praefurnium* (oven); and (v) sale: the establishment faced the street, where the baked bread was taken for sale [198,199] (Figure 6a).

The large presence of ovens and pastry shops for the production and sale of flour-based products suggests that baking was one of the most flourishing activities in Roman cities [199]. The larger establishments were almost always equipped with grinding wheels, a stable (the animals worked both in the milling of the cereal and in the transport of supplies and processed products when necessary) and in a residential area, but without a direct sales shop. The smaller establishments were more specifically dedicated to the sale of bread (*pistrina*, bakeries) and pastry (*pistrina dulciaria*, pastry-shops) [200].

Thirty-five *pistrina* have been identified in *Pompeii* (e.g., *House of the Chaste Lovers*, *Bakery of Popidius Priscus*) and another two in *Herculaneum* (e.g., *Bakery of Sextus Patulcius Felix*) [172,201], some of them equipped with stables where skeletal remains of equines have been found. Interesting is the *House of the Chaste Lovers* in *Pompeii*, the property of a rich baker named *Caius Iulius Polybius*. This house had an adjoining bakery with a splendidly preserved oven, millstones and two stables with the skeletal remains of five equids. Adjacent to one of the stables, a little flat with a closet was found, which was probably inhabited by the groom. The stable boy was in charge of cleaning the stable, the equids, giving them food, changing the bedding and all other activities connected with the stable [124].

In this way, working and consuming contaminated and deteriorated legumes and cereals entailed health problems for millers, bakers and consumers [193], as testified in the ancient sources as well as in the clinical findings, diagnoses and cures of many of the diseases described in medical treatises, such as Galen’s (*De Differentiis Febrium*, *De Alimentorum Facultatibus*; 2nd century CE) [191]. Warehouse workers, millers, bakers and others who handled grain were vulnerable to skin reactions, inhalant allergies and pathogens. The use of low-quality grain was also problematic, not only because it was less nutritious, but also because the consumption of bread made with spoiled grain could lead to health problems since pathogens were easily transmitted when people ingested insects or moulds that contaminated them [190,191]. In addition, the stable, mill and bakery were usually connected because they were part of the same bread-making process, so the close contact of humans with equids propitiated the ideal environment to acquire infections—parasitic, bacterial, viral and fungal—that could be transmitted by direct contact with these animals, their urine or excrement or by the consumption of legumes, cereals and bread that had been contaminated by coming into contact with the urine and excrement of these animals during handling and processing.

#### 4.4.5. Foodborne Zoonotic Diseases in Taverns (*Thermopolia*, *Cauponae*, *Popinae*) and Home

The complex theme of the diet in Roman times constituted and continues to present a fertile area of research, also in relation to the state of health and disease of ancient Roman populations. In fact, from the perspective of studies increasingly focused on a multidisciplinary approach, and therefore on cooperation between different disciplines such as archaeology, archaeozoology, archaeobotany, physical anthropology, classical literature and the history of Roman art [73], an attempt is being made to outline an ever more comprehensive picture both regarding the complex variety of food products available on the market and consumed by at least part of the Roman population, as well as the methods of preparation, handling and contamination of food. In particular, the issue of food contamination by pathogens is of considerable interest as it allows us to advance hypotheses about the state of health and disease of ancient populations in close relationship with eating habits. After all, it is not surprising how, in ancient times, the populations could suffer from different diseases associated with the consumption of contaminated food, as it is known that some foods were not subjected to adequate processing before their consumption and therefore could become possible vehicles for the transmission of zoonotic diseases. The sources from which to outline the eating habits of the Vesuvian region are numerous. Latin writers like Apicius (*De Re Coquinaria*, 1st century CE), Columella (*De Re Rustica*, 1st century CE) and Pliny the Elder (*Naturalis Historia*, 1st century CE) talk about Pompeian cooking. Their works offer a comprehensive idea of what should not be missing in the kitchens and tables of the people of *Pompeii*. Other evidence of priceless historical value comes from *Herculaneum*, *Oplontis* and *Stabiae*. These sites have yielded frescoes and mosaics showing convivial scenes, remains and residues of crops and charred remains of food in perfect condition of preservation.

No doubt, the culinary habits of the ancient Roman world deserve a more extensive approach from the sanitary point of view, since animals destined for food are the main reservoirs of many zoonotic foodborne pathogens, and food products of animal origin are the main transmission vehicles [12]. Foodborne zoonotic diseases are caused by the consumption of food contaminated by pathogenic microorganisms such as bacteria, viruses and parasites. The risks of contamination can occur at any point along the food chain: at the farm, during the slaughter and during processing or preparation and distribution [12]. It can also occur in taverns (e.g., *thermopolia*, *cauponae*, *popinae*) or in the home, if food is incorrectly handled or cooked (Figure 6b).

From *Pompeii* and *Herculaneum* comes a large quantity of zooarchaeological material associated with slaughtered bones and/or with burn marks that constitute tangible proof that meat was most frequently consumed on Roman tables, as well as providing information regarding the methods of handling (e.g., slaughter) and preparation (e.g., cooking). Although later than the time of the eruption, of notable interest is the *Edictum de Pretiis Rerum Venalium* (Edict Concerning the Sale Price of Goods), also known as Diocletian’s Edict on Maximum Prices, which was an edict promulgated by the Roman Emperor Diocletian that set maximum prices for more than 1300 products, as well as to establish labour costs to produce them [202]. According to the Diocletian’s Edict, the most affordable protein included sea fish, river fish, eggs, sheep’s milk and fresh cheese [202,203].

Meat sources included sheep/goat, beef, pork and poultry. In addition to these farm animals, the Romans also ate wild animals, such as wild boar, deer, wild donkeys, chamois, hare and birds, and other animals considered exotic such as flamingos and dormice [204] (see Section 4.3.2. Livestock for more details on the dormouse). However, the popular *farcimina* (spelt and meat sausages) were the cheapest meat and probably the unhealthiest since they combined low-quality ingredients with intense handling [81].

The excavation of an ancient sewer in the city of *Herculaneum* provided an opportunity to study the Roman diet in the Gulf of Naples, including marine resources used as food. The remains of human and kitchen waste that accumulated in the sewer—representing the waste of middle- and lower-class Romans that lived in an urban context—showed a high degree of dietary diversity, with around 70 fish, 48 marine molluscs and 3 identified marine arthropod taxa [64]. With regard to fish, the highest quantities found correspond to black seabream (*Spondyliosoma cantharus*), damselfish (*Chromis chromis*), followed by seven identified *Sparidae* species. Of the edible shellfish, the only species recovered in extremely large quantities were the corneous wedge clam (*Donacilla cornea*), limpet (*Patella* spp.) and purple sea urchin (*Paracentrotus lividus*) [205]. Both fish and shellfish would have been available locally in large quantities and probably represented cheap and nutritious dietary fare. Some of the fish consumed was likely salted (*salsamenta*) or fermented (such as fish sauces such as *garum*), but it is believed that most of the fish and shellfish originated in the Gulf of Naples and would have been purchased fresh [64].

Among all the foods that the Romans consumed and appreciated in a particular way, and which may have represented a food vehicle for the infection and transmission of intestinal parasites, there is certainly the *garum* [206]. The consumption and production of *garum*, a sauce obtained from the fermentation in brine of whole fish products or parts of them, including entrails and blood mixed with different spices, wine and vinegar, was also very frequent in *Herculaneum* and *Pompeii* [207]. Much of the evidence about this ancient fish sauce comes from classical literary sources (e.g., Pliny the Elder *Naturalis Historia* XXI.93ff, 1st century CE; Apicius *De Re Coquinaria*, 1st century CE). Furthermore, Pliny the Elder (*Naturalis Historia* XXI.94; 1st century CE) cites *Pompeii*, together with *Betica*, *Mauretania Tingitana* and *Leptis Magna*, as one of the most famous places of production and export of *garum* in the Mediterranean. Likely, *Pompeii* had great fortune in the *garum* production industry thanks to its privileged position in the Gulf of Naples and near the mouth of the Sarno river, where the supply of salt together with a wide range of fish resources must certainly have been abundant [207]. Generally, the processing of the fish used to obtain *garum* by salting took place in special production areas located in suburban areas, within which innumerable tubs or containers were used for maceration, as evidenced by the discovery of an industrial area linked to the processing of fish products near Porta Stabia at *Pompeii* [208]. The procedure for producing this famous sauce involved sun-drying fish between 2 and 3 months; however, there were cases in which maceration was accelerated by exposure to other heat sources such as ovens [209]. In *Pompeii,* the recent discovery of a ‘*garum shop*’, a building interpreted as a place of production of this sauce, not only allowed more in-depth zooarchaeological analyses to be carried out on the remains of the fish found still in a state of maceration in six perfectly preserved *dolia* at the time of the 79 CE eruption, but also the obtention of more information about the production techniques [210,211,212].

Both meat and fish infected or contaminated during their handling and preparation for consumption could transmit zoonotic diseases like those we see today if they were consumed undercooked, subjected to mild brines, or lightly smoked. Thus, diseases caused by *Mycobacterium* spp., *Campylobacter* spp., *Salmonella* spp., *Streptococcus iniae*, *Clostridium botulinum*, *Listeria monocytogenes* or *Vibrio vulnificus*, and some parasites, such as *Cryptosporidium* spp., *Balantidium* spp., *Taenia saginata*, *Giardia lamblia* or *Toxoplasma gondii*, would not have been rare [2,12,213,214,215]. Hippocrates (*Corpus Hippocraticum*, Book of Epidemics; 4th century BCE) even recommended cooking meat and seafood well to avoid diarrhoea [216].

Foodborne zoonoses acquired from meat are too many to mention, but perhaps bovine tuberculosis could be considered a probable zoonotic disease in Roman times. In fact, pathological skeletal changes to the ribs and vertebrae indicative of tuberculosis have been documented in two adults in the *Herculaneum* osteological sample [217]. Tuberculosis is caused by a bacterium that belongs to the genus *Mycobacterium*, and Capasso and Di Tota [217] suggested that it was possible that the disease may have been contracted by the consumption of undercooked infected oxen viscera after ritual sacrifice, though there were probably other sources of infection.

There are several literary sources that testify that in Roman times sheep farming was frequently cited not only in relation to the consumption of meat, but also to the production of wool, milk and its derivates (Varro *Res Rusticae* II.8, II.11.1, 1st century BCE; Columella *De Re Rustica* VII.2, 1st century CE; Pliny the Elder *Naturalis Historia* VIII.123–124, 180, 1st century CE). With regard to sheep’s milk, the Romans considered it an essential food in their daily diet (Varro *Res Rusticae* II.2.2, 1st century BCE; Pliny the Elder *Naturalis Historia* XXVIII, XXXIII, CXXIV, 1st century CE), so much so that even Celsus in his medical treatise recommended its intake, underlining its health benefits (Celsus *De Medicina*, 1st century CE). Particularly appreciated, as well as being considered a particularly delicious food, was the *colostrum* (the milk of a sheep that had just given birth). Further information and confirmation about the use of this food can be found in the work of Apicius (*De Re Coquinaria* VII, XI, XIII, 1st century CE). In his *corpus* of recipes, the author, among the various products handed down in the culinary field, mentions milk as a fundamental basis for the preparation of desserts, creams, cheeses and sauces. Speaking of sauces, the Romans used to consume *melca*, a kind of yoghurt or sour cream obtained by using sour milk mixed with different spices, such as pepper, salt, oil, coriander, old-fashioned *melca*, herbs and onions [218,219]. More generally, fresh and unpasteurized raw milk, mixed with fig juice regurgitated by young lambs not yet weaned, was used to produce fresh cheeses (Pliny the Elder *Naturalis Historia* XI.239, 1st century CE). Fresh cheeses were usually intended mostly for quick consumption, unlike those that were seasoned with the aid of salt. In this case, according to what is always learned from literary sources (Varro *Res Rusticae* II.2.3, 1st century BCE; Columella *De Re Rustica* VII.2.1, VII.8.1, 1st century CE), which accurately describe the production procedures of fresh and aged cheeses, there were cases in which dairy products were preserved by previous drying in the sun and subsequent pressing and immersion in brine [207,220]. In central and southern Italy, the milk, if not used for the preparation of cheeses, was eaten fresh during the day or curdled with the addition of aromatic herbs given the difficulty of preservation.

Renowned in coeval sources for the production and consumption of sheep and goat milk, the ancient Vesuvian city of *Herculaneum* has given us back several portions of charred cheese, whose microbiological examination has allowed us to infer information about how these foods were consumed and used in Roman gastronomy, as well as in relation to their processing methods. In fact, inside one of these charred cheeses was found the presence of spherical-shaped bacteria that, both in terms of morphology and dimensions, seemed to be consistent with the genus *Brucella*. The heat of the eruption, coupled with the subsequent burial environment, made it impossible to use molecular techniques to confirm identification [61]. Moreover, Capasso [221] described vertebral lesions associated with rib alterations in 16 adult individuals of the *Herculaneum* skeletal sample, which suggested brucellosis. The high frequency of the disease (about 17.4% of the adults) is also supported by historic evidence (Varro *Res Rusticae*; II.5.4, 1st century BCE; Cicero *De Natura Deorum* II.159, 1st century BCE; Pliny the Elder *Naturalis Historia* VIII.180, 1st century CE) and the carbonized remains of cheese. Thus, the historical and biological data from the *Herculaneum* population provides the first evidence of a relationship between milk-related sources and brucellosis.

In Roman cuisine, it was also common to use and consume other foods of animal origin through which it was possible to contract some zoonotic diseases. Among these were, for example, the eggs, not only of hens but also of goose, ducks, peacocks and wild birds. Eggs were a widely frequent food in Roman gastronomy. Apicius (*De Re Coquinaria*, 1st century CE) reports their use in various recipes as a basis for the preparation of sauces, binders and desserts. Among the countless organic remains found in *Herculaneum*, eggs were also among the foods contaminated by pathogens that may have negatively affected the health of the population. As in the case of cheese, the microbiological examination performed on the interior surface of the preserved charred eggshell of hard-boiled eggs from *Herculaneum* also showed the presence of a cocciform bacterium morphologically and dimensionally consistent with the genus *Salmonella* [62]. In the past, as in modern times, salmonellosis could be transmitted to humans via the faecal—oral route, that is, either through the ingestion of contaminated foods, such as in the case of eggs, or through the contact and/or handling of objects soiled by the faeces of small domestic animals affected by *Salmonella* [78,222]. Therefore, infections in ancient Roman populations could be contracted both during the collection of the eggs, such as through the use and manipulation of these during the preparation of some dishes that required their use [222]. Albeit in a risky way, even in the case of eggs, it can be assumed that these could have constituted a food that could have acted as a vehicle for the diffusion of zoonosis in humans [78].

### 4.5. Latrines, Sewers and Baths (Thermae)

In some houses or in certain corners of the *insulae* could be found *sterquilinia* (private latrines), located next to the kitchen or even inside it. This disposition was understood to easily get rid of organic waste and excess liquids since the number of pipes in the houses was reduced, thus simplifying and making their construction cheaper; however, the risk of food contamination in such combined kitchen/latrine areas must have been very high [85]. In addition, these latrines were not usually cleaned with water, nor were they connected to the municipal sewer system, but instead led directly to cesspits built with porous stone and without mortar, which allowed the drainage of liquids, leaving the solid residue to be cleaned periodically [85,200]. It is believed that this disconnection of the latrines from the sewer system could be due to avoiding possible backflows and accumulations of gases, evading the mandatory payment of the right to use the sewage system or preventing the entry of animals and insects from the sewers. However, it seems clear that the most likely cause was the value that faeces had as manure from both animal and human waste [167]. According to the written sources (Martial *Epigrammata* I.37.1–2, 1st century CE; Petronius *Satyricon* XXVII.3, XLI.9, XLVII.5, 1st century CE), urinals of various types also abounded, such as the *matellae* and the *lasana*—vessels for the reception of human urine and excrements, which the slaves (known as *lasanophoroi*) emptied into the latrines [85,223].

On the other hand, the *foricae* (communal latrines) seemed to be a flourishing business, which had jugs located in the streets where, for a modest tax, it was possible to urinate [85]. Lucretius (*De Rerum Natura* 4.1026–1029, 1st century BCE) refers to the use of vessels named *dolia curta* as a urinal/urine container—these containers were terracotta *amphorae* with their upper portion removed [179,224]. The *foricarii* and *stercorarii* were in charge of keeping the latrines, sewers and urinals clean, as well as transporting in a *plaustrum*—an open wagon or cart for hauling loads—the waste from the city to rural districts, where they were sold to farmers as manure [162]. Columella (*De Re Rustica* I.6.24, 1st century CE) already mentions that human faeces were recycled into fertilizer for crops (see Section 4.4.1. Agriculture), so that an entire infrastructure had been developed for its use. In addition, urine was used as a detergent in the *fullonicae*, establishments dedicated to the cleaning of fabrics and wool; see Section 4.4.3. Textile industry: Laundries (*fullonicae*): as a mordant for some dyes in the *officinae infectoriae*, workshops specializing in the dyeing of new textile products; in the *officinae offectoriae*, workshops for the dyeing of used textile products; and as a bleach in *officinae tinctoriae* [173]. Hence, the installation of *dolia curta* on public roads to be able to collect it [224].

Cesspits retained their popularity into the 2nd century CE, well after public toilets, flushed by sewer drains, were firmly established in the urban fabric. The persistence of cesspits probably had as much to do with simple economics than with resistance to change. A city notice found at *Herculaneum* (*exemta ste(r)cora a(ssibus) XI*) illustrates that 11 donkeys might have been paid for the removal of manure [74] (p. 101). *Foricarii* and *stercorarii* cleared it from the streets but also emptied cesspits and sold the contents to farmers on the outskirts of the city. The 11 donkeys mentioned in the *Herculaneum* inscription was not a very substantial sum, but when that sum was added to a farmer’s payment for the manure, it can be seen as being of modest economic value for the poorer urban dweller who was willing to take on this odious task [85].

**Figure 7:**
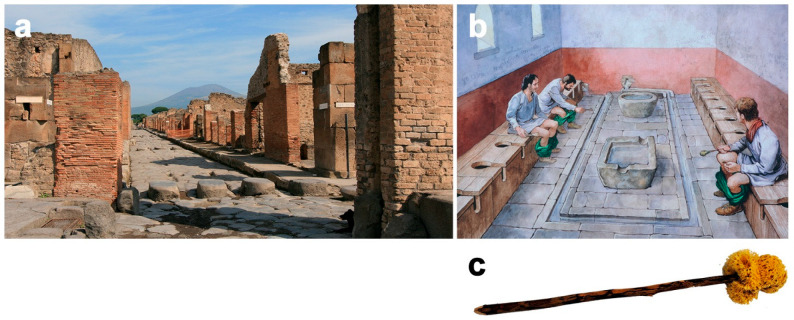
(**a**) Stepping-stones in Via *Stabiana* (*Pompeii*), whose function was to keep Romans’ feet dry and out of the rainwater, slop and animal waste that would have filled the streets [Author: Jensens. Distributed under a CC0 license. Available online: https://cutt.ly/aRCZ8V8. Accessed: 30 October 2021]. (**b**) Reconstruction drawing showing the communal latrines in use [Author: Carole Raddato. Distributed under a CC BY-SA 2.0 license. Available online: https://cutt.ly/dRCZ471. Accessed: 30 October 2021]. (**c**) Replica of *xylospongium* (sponge on a stick). It was a hygienic tool used and shared in communal latrines by ancient Romans to wipe their buttocks after defecating [Author: D. Herdemerten. Distributed under a CC BY 3.0 license. Available online: https://cutt.ly/iRCZ7GO. Accessed: 30 October 2021]. All images are of public domain via Wikimedia Commons webpage.

The problems associated with the disposal of human waste could be of a different order or nature, starting from the emanation of bad smells that acted as an attractive element for some animals, which, coming into contact with infected material, became, in turn, a vehicle for the transmission of pathogens moving freely around the city [85,167]. Generally, in Roman cities, the disposal of excrement and waste was closely linked to a complex system of public sewers, both public (*foricae*) and private (*sterquilinia*) latrines or simple cesspits located near the house that facilitated the unloading and drainage of wastewater. Despite the rigorous norms of Roman sanitary hygiene legislation, it was not mandatory to be connected to a public sewage system since this was a paid service [85]. For the houses or shops that were not connected, there was a private service in which the *foricarii* and *stercorarii*—generally people of lower social status, mostly prisoners and slaves—came to the building every day to collect waste from clay containers, the content of which was sold to farmers outside the city [85,162,163]. However, the conditions varied throughout the Empire. For example, in *Pompeii*, buckets containing waste and faecal matter were thrown out of the windows directly onto the street below, where they accumulated and dispersed, favouring the spread of diseases. The installation of stepping-stones documents this fact so that people could cross the street without getting their feet wet [225] (Figure 7a). To keep the streets clean, the *aediles*—the town authorities—of *Herculaneum* established a series of prohibitions and orders, such as a ban on throwing garbage on the ground near public drinking fountains. In an information panel located directly near the fountain, the *aediles* established sanctions for this violation: citizens had to pay a fine, and slaves had to be flogged [225,226]. However, even the enviable sewers did not guarantee complete health since their maintenance could be insufficient. For example, Caligula punished Vespasian—still a mere mayor—for not keeping the streets and sewers clean, and Julius Caesar forced citizens to take care of cleaning their streets [225]. In addition, *foricarii* and *stercorarii* were exposed to rats, insects and wastewaters, which frequently stagnated, and at times the same rainwater filled the sewers and cloacae and carried excrement and debris to the surface [85]. It is thus clear that there were numerous health implications as a consequence of the reuse of human faeces and urine, such as (i) plants fertilized with human—and animal—manure and converted into a probable source of pathogens; (ii) losses during transport in porous containers—such as the *dolia curta*, which were made of terracotta—and in the *plaustra*—obviously these carts were not watertight; (iii) handling of urine and faeces by *foricarii* and *stercorarii*; (iv) dunghills and the accumulation of faeces and urine prior to transportation; (v) contamination of waterpipes; or (vi) presence of puddles in the absence of adequate drainage systems [85].

More than 88 latrines have been discovered in the portion of *Herculaneum* that has been excavated. Out of 41 houses, 28 of them had one or more latrines, producing a total of 39. Of these, 14 were located next to a kitchen, while the rest were set in the servants’ quarters or service rooms. Latrines have also been found in many shops (*tabernae*) and workshops. Of the 68 commercial premises at *Herculaneum*, 27 had a toilet near the entrance, which might have benefitted not just the workers but also their clients [227]. In the case of latrines inside the kitchens or *tabernae* (shops selling foodstuffs), poor management of human waste disposal could pose a significant risk of accidental contamination of food with pathogens [75,77,170]. Finally, although the *forum* of *Herculaneum* has never been excavated and its various public buildings have only been partially uncovered, 11 public buildings were discovered, in which 9 latrines have been found [227]. It is interesting to note that the *foricae* (public latrines) were sometimes attached to *thermae* (baths) [162]. In the case of *Herculaneum*, four of the public latrines were in the Central Baths and one in the Suburban Baths [227].

The use of *thermae* and public latrines was a very widespread and common practice in the Roman custom that the entire population enjoyed daily. In particular, the *thermae* did not only serve as sanitation facilities but were also a place for social gathering and conversation [167] (Figure 7b). In the case of public latrines, these represented an environment in which it was possible to come into contact with parasites that could have negative health consequences on people’s health. The *xylospongium* or *tersorium*—also known by the sources as a hygienic tool used and shared in public latrines by the Romans—was a sponge fixed to a stick used to clean the buttocks after defecation and may well have been one of the vehicles responsible for the spread of parasites and intestinal tract bacteria in humans [167,228,229,230] (Figure 7c). Both Seneca (*Epistulae morales ad Lucilium* VIII.70.20, 1st century CE) and Martial (*Epigrammata* XII.48.7, 1st century CE), even if they do not describe the method of use, imply the context of use, which denotes the use of *xylospongium* in ancient latrines [85]. Moreover, widespread in the Roman world could have been the custom of not following strict hygiene rules regarding hand washing after using the latrines and after preparing food; in this case, this practice would suggest a further form of contamination of the food that was then ingested. This is especially the case in the consumption of fruit and vegetables, which did not always require a specific cooking method useful for killing any pathogens [167]. Like latrines, the *thermae* (baths) would have represented another environment potentially suitable for the proliferation and spread of zoonotic diseases because some zoonoses are also waterborne [231]. In the case of the *thermae*, it is possible that the water was not changed regularly and that consequently, this aspect led to an accumulation of waste and at the same time created, through the stagnation of dirty water and heat, an environment particularly suitable for the proliferation of parasites, such as roundworms and protozoa, which in turn infected humans causing gastrointestinal disorders, but some other vectorial diseases (viral and bacterial), including zoonoses like leishmaniosis could have been present.

In order to better understand the close relationship between dietary diversity, strategies for waste disposal and problems related to the public health of the population, once again is interesting the analysis of biological material from the *Cardo V* sewer at *Herculaneum*. This biological material includes human excrement and the remains of edible foodstuffs, including eggshells, seashells, fish bones, otoliths and botanical material. In general, this sewer lacked an outflow point and instead functioned as a cesspit to collect the human and kitchen waste generated by those that lived in a mixed commercial and residential structure whose shops and apartments were situated above (in the *Insula Orientalis II*) [64,65,205]. Several studies have been carried out on the faecal material coming from the latrines of *Herculaneum*, which confirm the presence and diffusion of both various intestinal parasites and of their eggs due to the contamination of food and water by human faeces; among the most common predominate the whipworm (*Trichuris trichiura*)*,* roundworm (*Ascaris lumbricoides*) and *Entamoeba histolytica*, which causes dysentery [75,206,232]. In a recent study, Ledger et al. [233] also observed helminth eggs of *Trichuris trichiura* and *Ascaris lumbricoides* when analysing pelvic sediment samples from skeletal remains from *Oplontis*. All these aspects would suggest how, despite the presence and use of public latrines being quite widespread in the Roman Empire, together with a complex system of washing plants, sewage networks and drinking water conveyed by aqueducts within the city, and even though hygiene rules and the disposal of human waste were not neglected, still these measures were insufficient to protect the population from the possibility of contracting parasites spread through faecal contamination.

### 4.6. Rituals

For the ancient Romans, animal sacrifice was performed as a ritual to communicate with gods, heroes and other divine beings. Such rituals were intended to request favours, protection and help from divine recipients or to appease them through prayer. The sacrifice of animals was also a way for human worshippers to know the will of the gods, and the ritual often concluded with the distribution and consumption of the meat, which served to strengthen and define the social fabric by marking who belonged to a particular group and who was an outsider, expressed largely by the degree of access to meat [234]. The main evidence available for the study of ancient animal sacrifice is provided by literary texts, inscriptions, frescoes and archaeological remains in the form of altars and other sacrificial facilities, as well as animal skeletal remains [235,236,237,238]. These lines of evidence show that the animals chosen for sacrifice were generally of domestic species, such as cattle, sheep, goats or pigs.

In consideration of the use of livestock animals for ritual purposes, it is possible to hypothesize how this practice could have represented a further channel of transmission of zoonotic diseases. In fact, following the sacrifices, the animals were eviscerated primarily to make the offering to the gods (e.g., liver, heart, lungs, spleen, intestines) and subsequently to examine the contents of the internal organs to interpret the liking of the gods themselves [234]. Following the public sacrifice, the meat was distributed and served among those who had taken part in the sacrifice itself, and in some cases, to the population. In order for the worshippers to be able to consume the meat, the viscera and other remains of the animal, they first had to be returned to the profane sphere—in order to deprive them of a certain sacredness—which was done by boiling the meat in milk or simply in water, or the sacrificer placing his hand on the carcass, a gesture that transformed the meat into something that men could eat. From this moment, the meat could be divided and distributed [234]. The meat was often eaten in the sanctuary where the sacrifice had been, but it could also be carried in small *sportulae*—food baskets distributed as gifts by wealthy and influential people to clients and protégés [87] (p. 209)—to consume at home or sell in the *macella*—public markets dedicated to the sale of meat and fish [239].

It is evident that sacrificial meat was an important source of protein in the diet in ancient times, and meat consumed at public banquets appears to be derived from ritual sacrifices [240]. In the Roman *macella*, wild birds (e.g., pheasants and pigeons) and fish were sold, in addition to the most common meat of beef, lamb and pork [241,242]. Recent studies of the *macella* have shown that these facilities offered meat from both shrine sacrifices and from animals sacrificed in the market building, which was equipped with altars and statues of deities [239]. This situation is very evident in *Pompeii*, where the *macellum* is in the centre of the *forum*, which allows easy access to this open space, and in the vicinity of which there were a series of *tabernae* (shops selling foodstuffs) and more than ten temples. The animals sacrificed in these sanctuaries could have been sacrificed and sold in the *macellum*, as seems to be evidenced by the discovery of a small facility containing the bones of animals that died during the eruption of Vesuvius [234,243] (p. 467).

## 5. Concluding Remarks

There is no doubt that the cultural and urban environments contributed to the animal–human interaction in the daily life of the ancient Roman cities of *Pompeii* and *Herculaneum*. As testified in ancient medical (e.g., Celsus’ *De Medicina*; Galen’s volumes) and veterinary (e.g., Vegetius’ *Digesta Artis Mulomedicinalis*) treatises, numerous diagnoses and cures in relation to zoonotic diseases were outlined. Summarising, the main drivers and mechanisms for the distribution and transmission of zoonotic diseases could have been: (i) the large number of and the role played by different animal species in the ancient Roman world (e.g., livestock, wild, exotic and household animals); (ii) the environmental conditions for the survival of parasites, pathogens and vectors (e.g., population density, substandard housing, different available materials for the construction of the houses, use of animal and human wastes as fertilizer, lack of cleaning and hygiene); (iii) the great variety and intensity of commercial activities and occupations that represented certain risks of infections due to the continuous contact with animals and their products (e.g., agriculture and farming, textile industry, laundries, bakeries); (iv) the absence of adequate safety controls during the processing, distribution and preservation of foodstuffs in unsuitable environments, and some culinary habits (e.g., incorrect handling and cooking); (v) the inadequate mechanisms of the disposal of human waste and the biotic contamination of watercourses and reservoirs (e.g., combined kitchen/latrine areas, shared use of baths and public latrines); and (vi) the use of animals related to common cultural and religious practices (e.g., participation in public entertainment games, sacrifice as offerings to the gods).

## Figures and Tables

**Table 1:** Total area, population density and estimated population of *Pompeii* and *Herculaneum* compared with other major ancient cities of the Roman Empire.

Site	Modern Country	Total Area (ha)	Population Density (People per ha)	Estimated Population
*Roma*	Italy	1783	518	923,406
*Alexandria*	Egypt	972	422	410,535
*Antioch*	Turkey	399	313	124,936
*Carthage*	Tunisia	343	298	102,079
*Athens*	Greece	225	429	96,429
*Ephesus*	Turkey	263	272	71,587
*Lugdunum*	France	170	357	60,714
*Londinium*	United Kingdom	160	250	40,000
*Ostia*	Italy	154	227	35,017
*Neapolis*	Italy	82	275	22,550
*Verulamium*	United Kingdom	90	183	16,500
*Fregellae*	Italy	80	125	10,000
*Pompeii*	Italy	60	166	9938
*Emerita Augusta*	Spain	81	120	9720
*Volubilis*	Morocco	43	211	9058
*Calleva Atrebatum*	United Kingdom	45	80	3600
*Verona*	Italy	52	68	3525
*Augusta Praetoria*	Italy	41	83	3417
*Italica*	Spain	49	65	3178
*Iulia Valentia Banasa*	Morocco	15	183	2738
*Herculaneum*	Italy	20	115	2290
*Luna*	Italy	23	80	1840
*Conimbriga*	Portugal	23	66	1519
*Emporiae*	Spain	21	63	1313

ha, hectare.

## Data Availability

Not applicable.
